# Supplementary material for: The burden of traumatic brain injury from low-energy falls among patients from 18 countries in the CENTER-TBI Registry: A comparative cohort study
Source: PLoS Med. 2021 Sep 14;18(9):e1003761. doi: 10.1371/journal.pmed.1003761 (PMC8509890; doi:10.1371/journal.pmed.1003761)
Supplement: S1 Analysis Plan — (DOCX) [file pmed.1003761.s001.docx]

The aim of this paper is to describe the demographic, injury and clinical characteristics of high energy and low energy TBI patients who presented to 56 CENTER-TBI recruiting hospitals in Europe and Israel including patients discharged from the Emergency Room (ER) after imaging.

**Methods**

We established a CENTER-TBI registry in 61 participating centres across 18 countries in Europe and Israel. However, 4 centres did not enrol any patients at the end of the recruitment period and 1 centre enrolled only 1 patient with missing pathway information. These centres were excluded from the analysis. Therefore, this report is based on 56 centres across 18 countries. Patients were enrolled into the CENTER-TBI registry between 01·12·2014 and 31·01·2018.

**
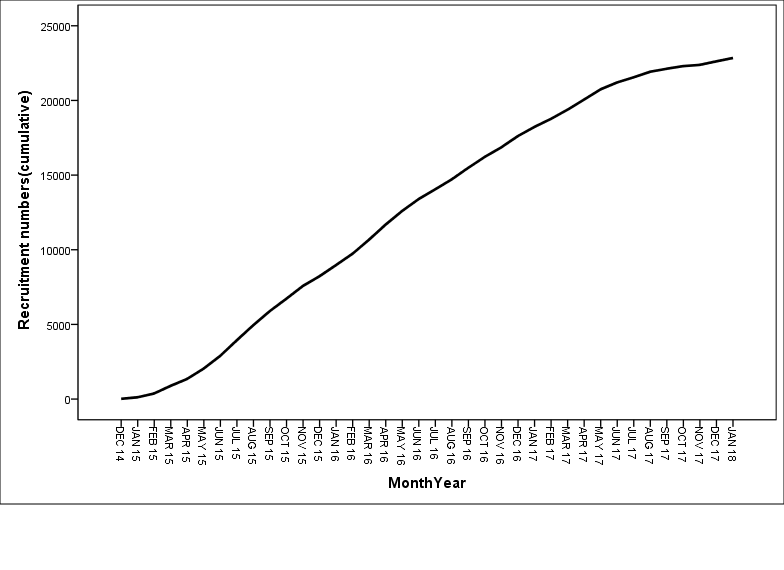
**

Figure 1: Cumulative sum of recruitment across 61 sites each month

**Inclusion and Exclusion Criteria**

There were no exclusion criteria for recruitment into the CENTER-TBI registry in patients with suspected or clinical TBI diagnosis as well as indicators for computed tomographic brain scan (CT). In particular, patients with pre-existing cognitive impairment such as dementia were included in the registry, as also those dying in the ED prior to imaging.

**Data collection procedures for the CENTER-TBI registry**

56 participating centres across Europe and Israel prospectively collated clinical data elements for TBI patients meeting the inclusion criteria of the CENTER-TBI registry over a 36-month period. No specific study interventions were performed. The internal logistics for data entry varied by site, with some sites performing direct web based data entry and others initially collecting a simple one-page case-report form in the ER over a 24-hour period, which was then entered by dedicated study staff the next day. Some sites extracted the registry variables from the clinical record after the patient had been discharged (or died). This registry methodology enabled data from several eligible patients to be collected in batches to increase efficiency and reducing cost. Sampling was purposive to include a representative sample (in terms of hour/day/month of presentation to hospital) of TBI patients.

**Research Government Approvals**

Informed consent was not required as no patient identifiers were retained – however national IRB approvals were sought as per national guidelines. For example, within the UK, approval was obtained from the national “Health Research Authority” (HRA).

**Case Report Form (CRF) Variables**

The CRF used by the participating sites to collect data contains variables describing demographics (age & gender), pre-existing health status, mechanism of injury, injury severity descriptors (Glasgow Coma Scale, Abbreviated Injury Scale (AIS), Injury Severity Score(ISS), presenting physiological vital signs, CT Brain findings, processes of care, and immediate outcome of care in terms of status on discharge (Table 2 ). These variables were derived from the Utstein trauma template used for standard trauma registry collection across Europe, North America and Australasia .^16^ Using the Utstein trauma template, registry data was kept as simple as possible to facilitate Comparative Effectiveness Research analysis on structural parameters. A web based data entry format was also implemented.

| TABLE 1: CENTER-TBI Registry Variables |
| --- |
| 1. Age |
| 1. Gender |
| 1. Pre-Injury American Society of Anaesthesiologists Physical   Status (ASA-PS) |
| 1. Head Neck AIS |
| 1. Total Injury Severity Score |
| 1. Mechanism Of Injury |
| 1. CT scan findings |
| 1. Glasgow Coma Scale |
| 1. Key Emergency Interventions |
| 1. Status on discharge & Discharge destination |

**Classification of Patients**

TBI patients with the following mechanisms of injury: Motor vehicle accidents, collisions involving bicycles and motor cycles, pedestrians hit by cars, falls of >1m, assaults, sports accidents were classified as high‐energy TBI, and falls from a standing height, falls of less than 2m as low‐energy TBI.

**Data Processing**

Data were cleaned to identify errors, ensuring data quality and minimal impact on results.^17^.

**Statistical Analysis**

The analysis was based on the CENTER-TBI Registry data version 1.0, downloaded from a data management tool, Neurobot-TBI (https://center-tbi.incf.org/) on the 8^th^ of November 2018

We carried out descriptive analyses. Non- parametric/continuous variables (Age, GCS, Abbreviated Injury Scale (AIS),^18^ Injury Severity scores (ISS)^19^ are presented as medians and interquartile ranges (IQR) while categorical variables such as injury mechanism, gender, type of injury and survival status are presented as numbers and percentages. Chi-square test was used to compare categorical variables across the low and high energy TBI categories, while the non- parametric/ continuous variables were compared using the independent samples T- test.

Analysis was performed using IBM Statistical Package for Social Sciences (SPSS) version 23, Microsoft excel 2010 and RStudio (1.0.136).

**Role of the funding source**

The funding source (European Commission Framework 7) had no role in the writing, or interpretation of the manuscript and the decision to submit for publication. The corresponding author had full access to all the data in the study and had final responsibility for the decision to submit for application.

**Results**

A total of 21681 TBI patients presenting to 56 CENTER TBI hospitals - with clinical care pathway and injury mechanism data - were included for analysis. Clinical care pathway was either discharge from the Emergency department (ER), admission to the hospital after CT brain scan but not directly to the ICU (ADM), or admission directly to the Intensive Care Unit (ICU).

Figure 2 shows the allocation of patients according to energy transfer mechanism. Overall, (13059)60% of 21681 TBI patients evaluated in the Emergency department (ED) were high energy TBI and (8622)40% were low energy TBI.

1101Missing and Unknown mechanisms of injury

22782

22849

67 cases excluded for missing clinical care pathway data

Figure 2: Identification of TBI registry cases as high and low energy

|  | **OVERALL(% N)** | **High Energy TBI ( % N)** | **Low Energy TBI( % N)** | **P value** |
| --- | --- | --- | --- | --- |
| TOTAL(N) | **21681** | **60.2(13059)** | **39.8(8622)** |  |
| *Demographic characteristics*  Median age (IQR) | 55(32-75) | 42(25-60) | 74(56-84) | <0.001 |
| - Under 16 years | 2.7(579) | 3.7(488) | 1.1(91) | <0.001 |
| - 16-64 yrs | 59.0(12782) | 76.1(9934) | 33.0(2848) |  |
| - 65 years and over | 38.4(8317) | 20.2(2635) | 65.9(5682) |  |
| *Male* |  |  |  |  |
| 1. Overall | 60.8(13186) | 67.6(8833) | 50.5(4353) |  |
| 1. under 16 | 64.1(371) | 64.8(316) | 60.4(55) | <0.001 |
| 1. 16-64 | 69.1(8831) | 70.9(7044) | 62.7(1787) |  |
| (iv) 65 and over | 47.9(3982) | 55.9(1472) | 44.2(2510) |  |
| *Care Pathway* |  |  |  | <0.001 |
| ER | 42.8(9286) | 42.2 (5511) | 43.8(3775) |  |
| Admission | 37.9(8224) | 33.4(4365) | 44.8(3859) |  |
| ICU | 19.2(4171) | 24.4(3183) | 11.5(988) |  |
| *Pre-injury health status and medical history* |  |  |  |  |
| Pre-injury ASA-PS classification |  |  |  |  |
| - Normal healthy patient | 40.1(8688) | 55.8(7285) | 16.3(1403) | <0.001 |
| - A patient with mild systemic disease | 28.9(6266) | 26.0(3391) | 33.3(2875) |  |
| - A patient with severe systemic disease | 23.5(5105) | 12.0(1569) | 41.0(3536) |  |
| - A patient with Life-threatening disease | 2.4(523) | 0.9(116) | 4.7(407) |  |
| Anticoagulants | 11.9(2578) | 5.7(744) | 21.3(1834) | <0.001 |
| Platelet Aggregate Inhibitors | 11.4(2466) | 6.0(783) | 19.5(1683) | <0.001 |
| Both Anticoagulants and Platelet Aggregate Inhibitors | 1.7(370) | 0.9(120) | 2.9(250) | <0.001 |
| Patients with intracranial lesions taking anticoagulants | 11.7(580) | 6.5(207) | 20.9(373) |  |
| Patients with intracranial lesions taking Platelet Aggregate Inhibitors | 13.7(679) | 8.5(269) | 23.0(410) |  |
| Patients with intracranial lesions taking both anticoagulants and Platelet Aggregate Inhibitors | 2.3(114) | 1.2(37) | 4.3(77) |  |
| PATHOPHYSIOLOGY AT ADMISSION |  |  |  |  |
| GCS AT ED ARRIVAL (median(IQR)) * | 15(14-15) | 15(14-15) | 15(14-15) |  |
| Mild ( GCS 13-15) | 81.6(17702) | 78.6(10270) | 86.2(7432) | <0.001 |
| Moderate (GCS 9-12) | 3.8(830) | 3.7(480) | 4.1(350) |  |
| Severe(GCS 3-8) | 5.5(1195) | 6.7(879) | 3.7(316) |  |
| No Sum | 8.5(1835) | 10.5(1366) | 5.4(469) |  |
| Hypoxia on ED arrival | 1.3(287) | 1.4(183) | 1.2(104) | <0.001 |
| Hypotension on ED arrival | 1.3(292) | 1.7(227) | 0.8(65) |  |
|  |  |  |  |  |
| *Pupillary reactivity* |  |  |  | <0.001 |
| None Reacting | 2.5(552) | 3.2(419) | 1.5(133) |  |
| One reacting | 2.2(483) | 2.4(313) | 2.0(170) |  |
| Both reacting | 89.5(19409) | 89.5(11694) | 89.5(7715) |  |
| *Referral* |  |  |  | <0.001 |
| Primary referral- Arrived from injury scene | 87.5(18975) | 86.8(11330) | 88.7(7645) |  |
| Secondary referral – Arrived from another hospital | 12.4(2692) | 13.2(1720) | 11.3(972) |  |

TABLE 2: DEMOGRAPHICS, INJURY MECHANISM, CO-MORBIDITY AND PRESENTING PHYSIOLOGY

|  | **OVERALL(% N)** | **High Energy TBI( % N)** | **Low Energy TBI( % N)** | **P value** |
| --- | --- | --- | --- | --- |
| *CT characteristics* | 21489 |  |  | <0.001 |
| %(95%CI) Normal CT Report | 68.0(14743) | 66.5(8690) | 70.2(6053) |  |
| %(95%CI) Abnormal CT | 31.1(6746) | 32.4(4226) | 29.2(2520) |  |
| %(95%) of Abnormal CT = Intracranial lesion ** | 73.5(4959) | 75.2(3177) | 70.7(1782) |  |
| EDH small ** | 9.2(619) | 10.8(456) | 6.5(163) | <0.001 |
| EDH large* * | 3.5(239) | 4.1(175) | 2.5(64) |  |
| ASDH small** | 30.2(2036) | 30.1(1271) | 30.4(765) | <0.001 |
| ASDH large** | 14.6(983) | 12.3(520) | 18.4(463) |  |
| Contusions small ** | 36.3(2449) | 40.2(1698) | 29.8(751) | <0.001 |
| Contusions large** | 8.4(567) | 9.6(404) | 6.5(163) |  |
| Compressed basal cisterns** | 11.7(791) | 13.0(548) | 9.6(243) |  |
| Midline Shift** | 23.6(1592) | 20.9(883) | 28.2(709) |  |
| Sub-Arachnoid Haemorrhage** | 52.0(3509) | 56.7(2397) | 44.1(1112) |  |
| Median Head-Neck AIS (IQR) | 2(1-3) | 2(1-3) | 1(1-2) | <0.001 |
| Median Cervical Spine AIS(IQR) | 0(0-0) | 0(0-0) | 0(0-0) | <0.001 |
| Median ISS (IQR) | 9(4-17) | 9(4-20) | 6(3-12) | <0.001 |
| Median Extracranial ISS (IQR) | 4(1-8) | 4(1-10) | 2(0-5) | <0.001 |
| % (95%CI) Arrived Intubated | 8.4(1812) | 11.5(1496) | 3.7(316) | <0.001 |
|  |  |  |  |  |
| Key Interventions |  |  |  |  |
| % (95%CI) Had at least one Key emergency Intervention | 11.1(2397) | 13.4(1752) | 7.5(645) | <0.001 |
| % (95%CI) Key emergency Interventions performed |  |  |  |  |
| Craniotomy For Haematoma | 3.5(767) | 3.1(401) | 0.9(74) | <0.001 |
| ICP insertion | 3.5(753) | 4.7(609) | 1.7(144) | <0.001 |
| Decompressive Craniectomy | 1.3(275) | 1.5(201) | 0.9(74) | <0.001 |
| External Fixation Limb | 1.4(293) | 2.1(276) | 0.2(17) |  |
| ^Others | 2.1(445) | 2.8(369) | 0.9(76) |  |
| %(95%CI) survival to hospital discharge(alive) -destinations shown below | 93.3(20228) | 93.0(12146) | 93.7(8082) | <0.001 |
| Discharged home | 70.7(15324) | 72.4(9458) | 68.0(5866) |  |
| Discharged other hospital | 9.9(2151) | 10.3(1351) | 9.3(800) |  |
| Discharged to rehabilitation | 5.6(1221) | 5.9(776) | 5.2(445) |  |
| Discharged to nursing home | 5.2(1122) | 2.2(289) | 9.7(833) |  |

TABLE 3: IMAGING FINDINGS, INJURY SEVERITY, THERAPEUTIC INTERVENTIONS, TRANSFERS AND DISCHARGE STATUS

^OTHERS include External Ventricular CSF Drainage, Interventional Radiology, Damage Control Thoracotomy &Laparotomy, Extra peritoneal Pelvic packing. *Missing data is > 5·0%. ** denominator= those with abnormal CT

Figure 3: Comparison of patients with high and low energy TBI by age group (children 0-15years, younger adults 16-64 years, older adults >64 years).

Figure 4: Comparison of patients with high and low energy TBI by Care pathway (children 0-15years, younger adults 16-64 years, older adults >64 years).
