# Supplementary figures and images for: The burden of traumatic brain injury from low-energy falls among patients from 18 countries in the CENTER-TBI Registry: A comparative cohort study
Source: PLoS Med. 2021 Sep 14;18(9):e1003761. doi: 10.1371/journal.pmed.1003761 (PMC8509890; doi:10.1371/journal.pmed.1003761)

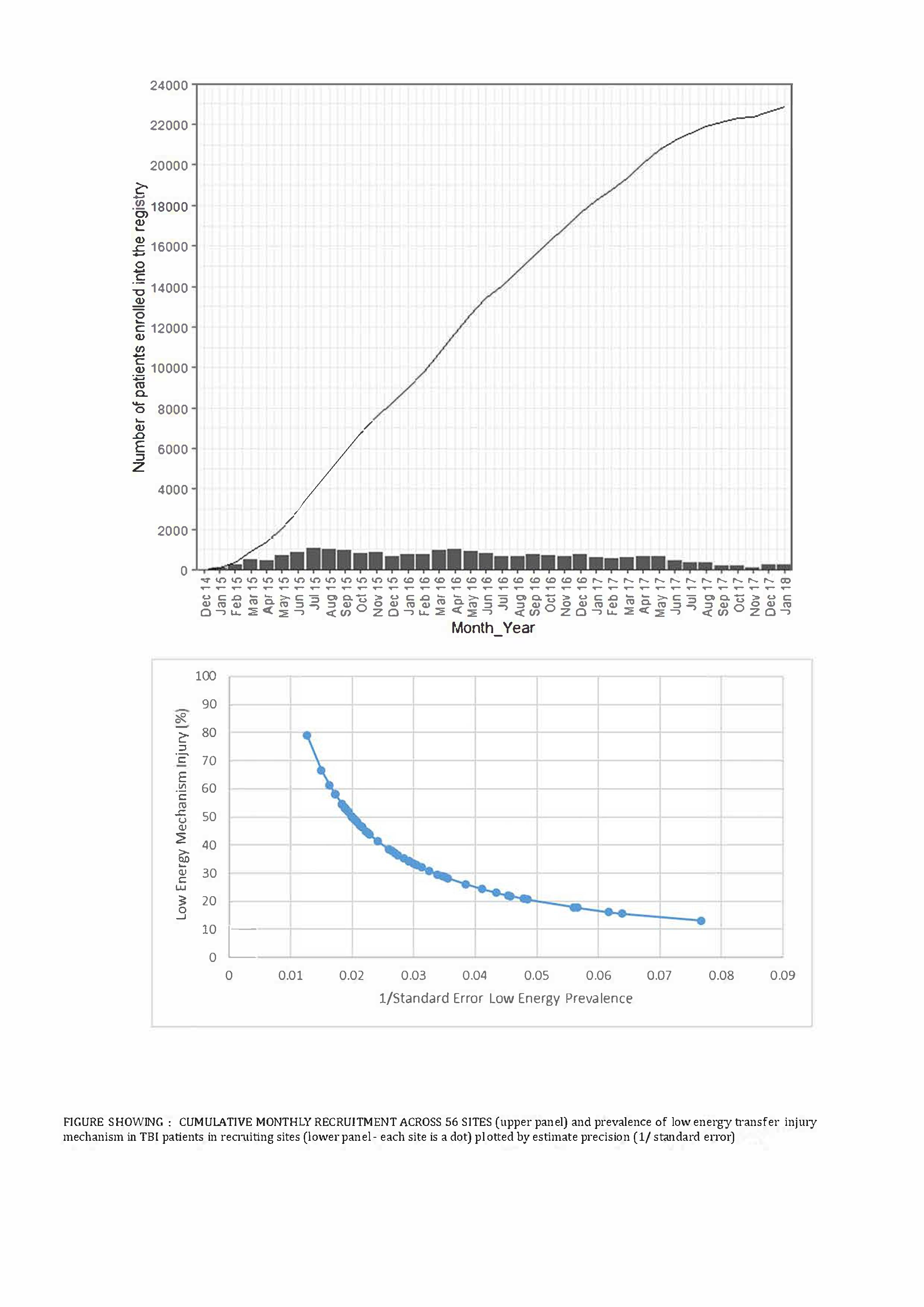

Supplement: S1 Fig — (TIF) [file pmed.1003761.s002.tif]

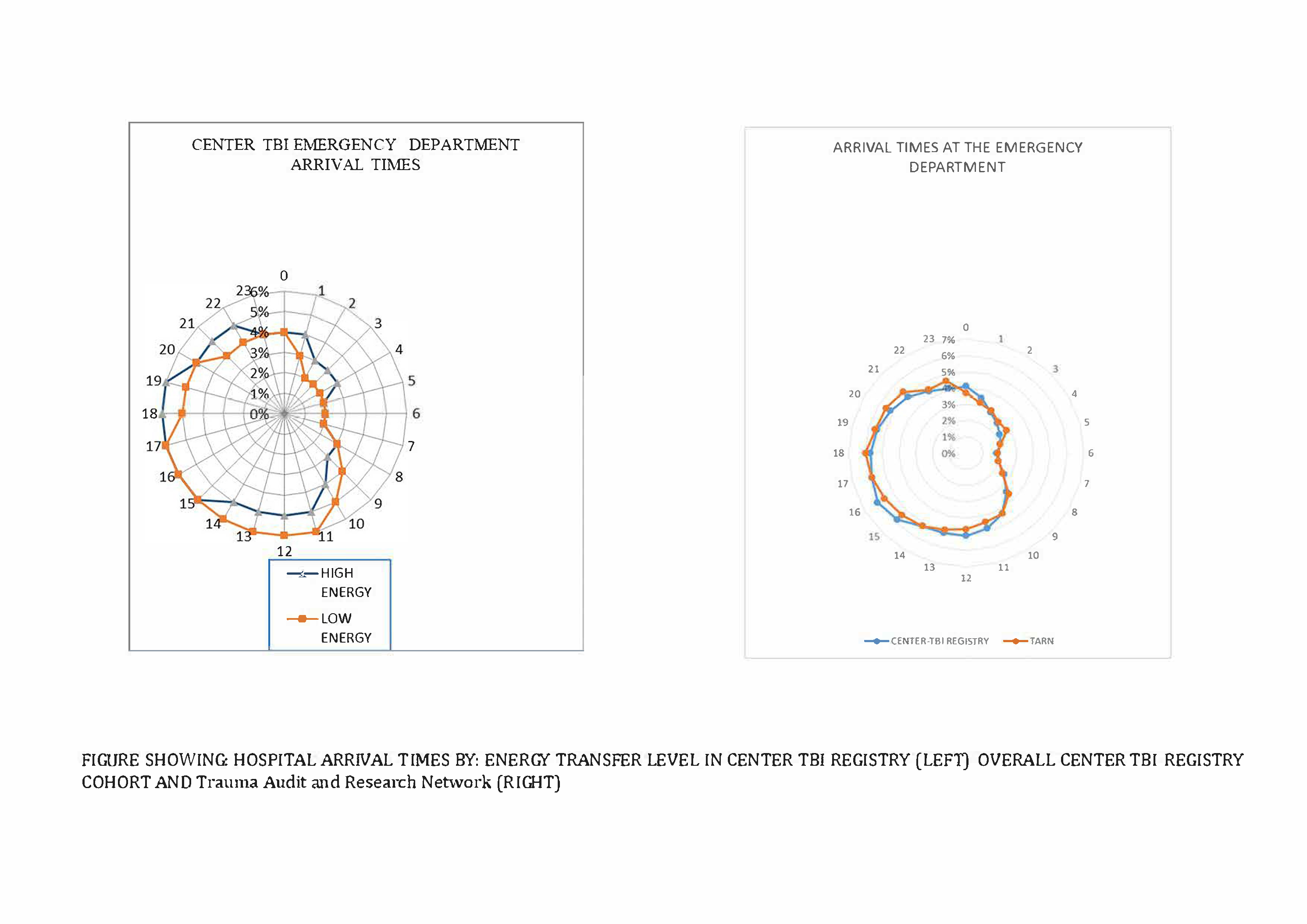

Supplement: S2 Fig — (TIF) [file pmed.1003761.s003.tif]

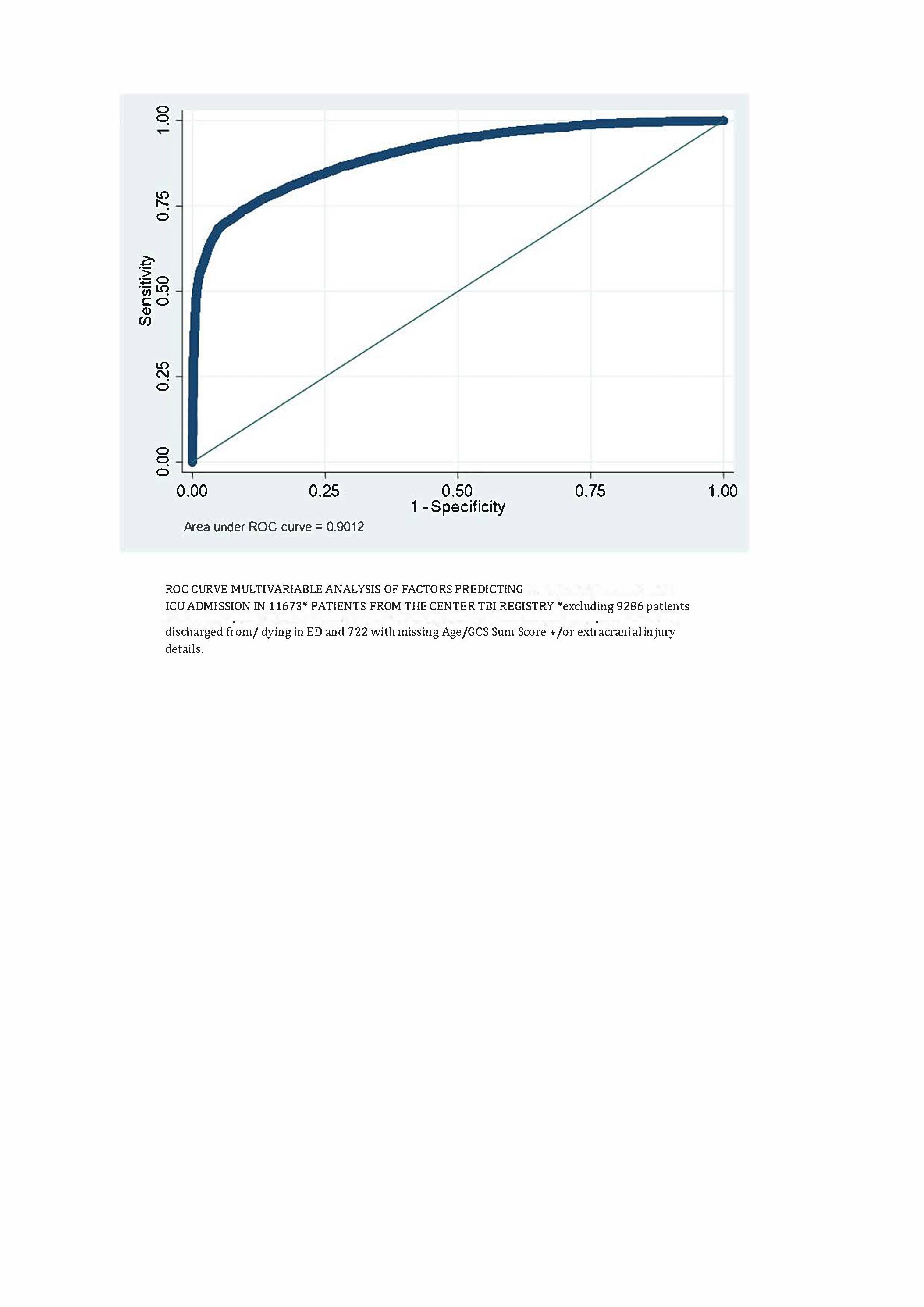

Supplement: S3 Fig — *Excluding 9,286 patients who were discharged from or died in the ED and 722 with missing age/GCS sum score and/or extracranial injury details. (TIF) [file pmed.1003761.s004.tif]

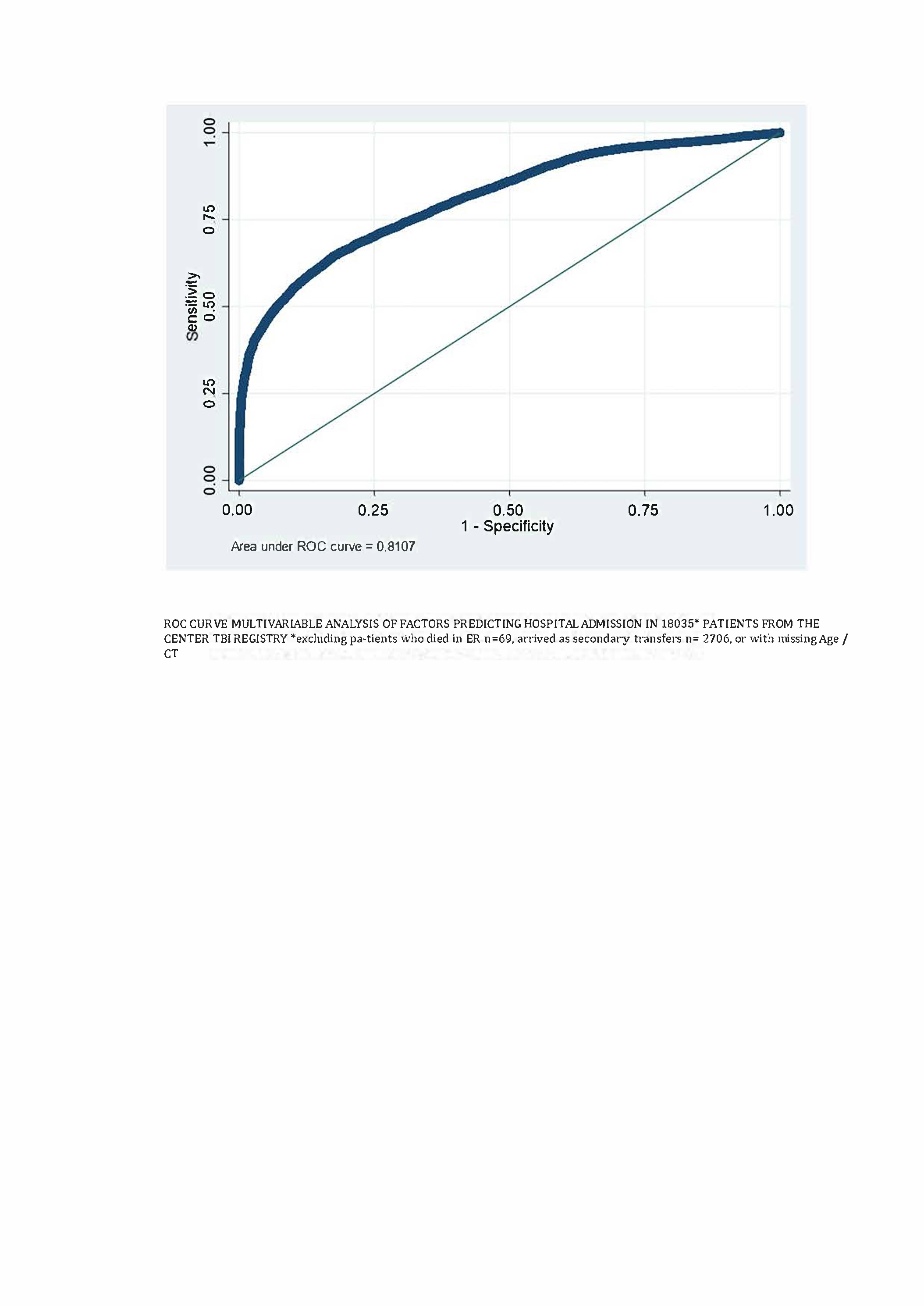

Supplement: S4 Fig — *Excluding patients who died in the ED (n = 69), arrived as secondary transfers (n = 2,706), or had missing age/CT. (TIF) [file pmed.1003761.s005.tif]

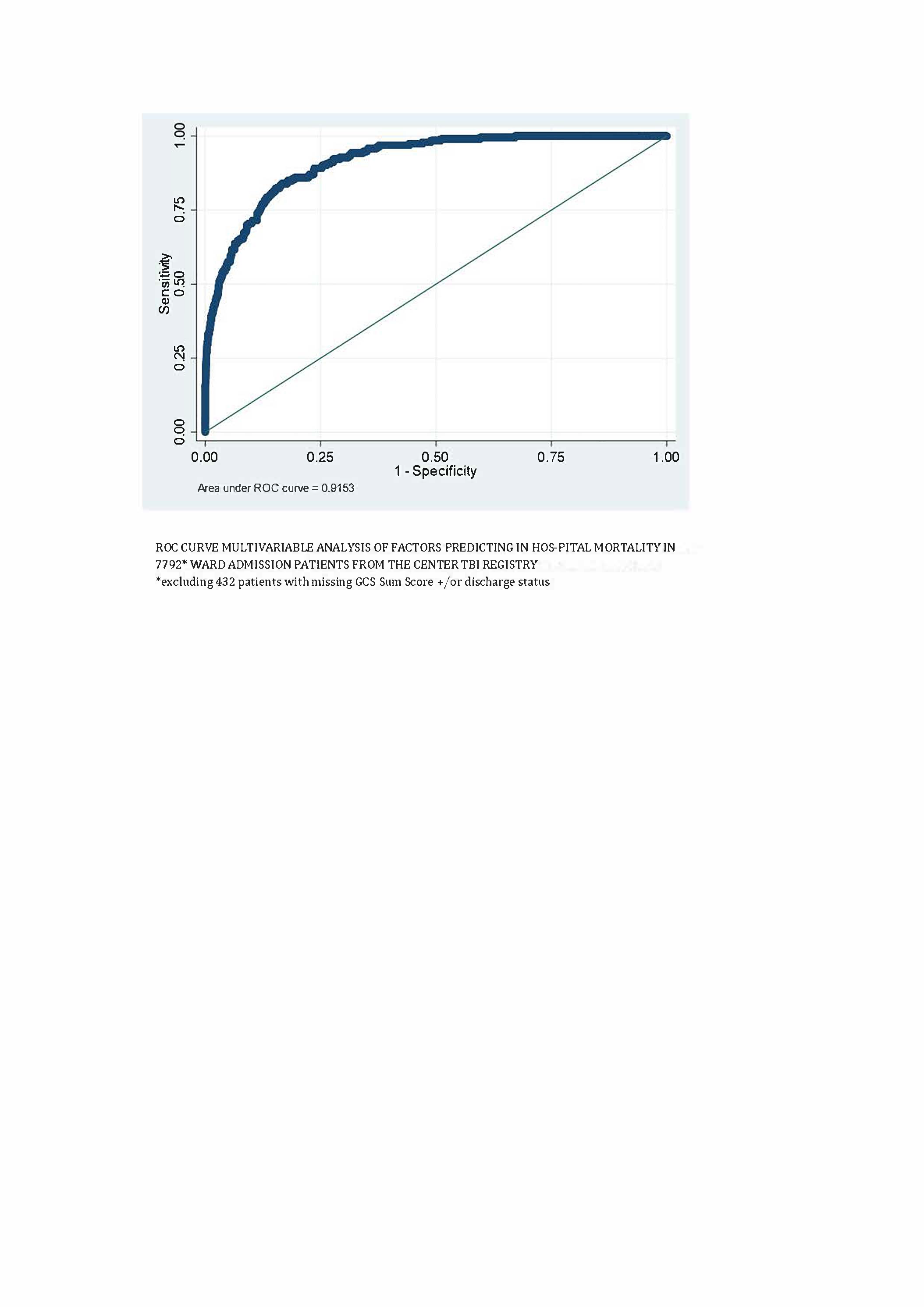

Supplement: S5 Fig — *Excluding 432 patients with missing GCS sum score and/or discharge status. (TIF) [file pmed.1003761.s006.tif]

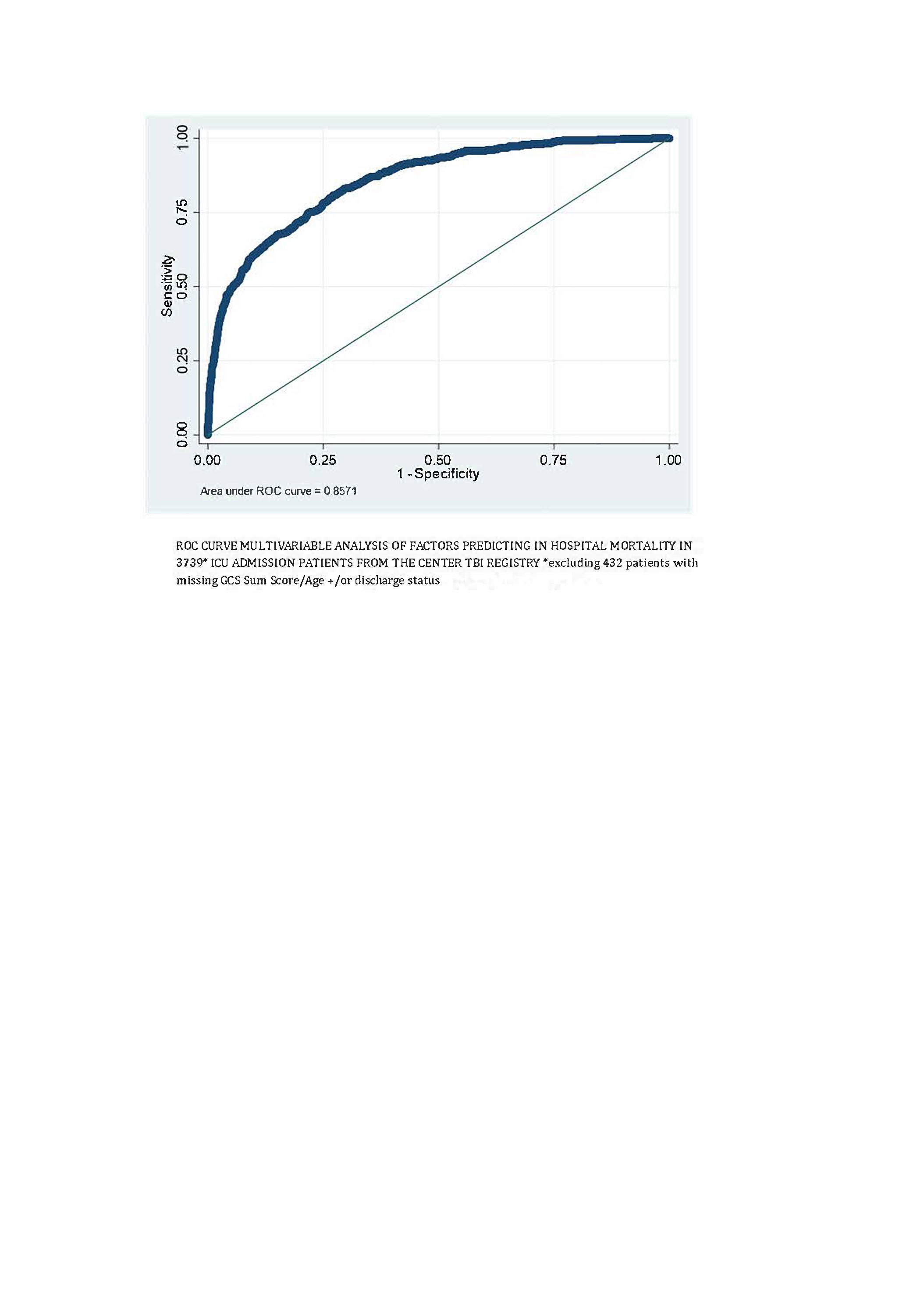

Supplement: S6 Fig — *Excluding 432 patients with missing GCS sum score/age and/or discharge status. (TIF) [file pmed.1003761.s007.tif]
