# Supplementary material for: The burden of traumatic brain injury from low-energy falls among patients from 18 countries in the CENTER-TBI Registry: A comparative cohort study
Source: PLoS Med. 2021 Sep 14;18(9):e1003761. doi: 10.1371/journal.pmed.1003761 (PMC8509890; doi:10.1371/journal.pmed.1003761)
Supplement: S1 Table — (DOCX) [file pmed.1003761.s009.docx]

| **Hospital** | **City** | **Country** |
| --- | --- | --- |
| Oslo University Hospital | Oslo | Norway |
| University Hospital Norther Norway | Tromso | Norway |
| University Hospital Nancy | Nancy | France |
| Raffaele University Hospital | Milan | Italy |
| Radboud University Medical Center | Nijmegen | The Netherlands |
| Medical University of Innsbruck | Innsbruck | Austria |
| Karolinska University Hospital | Stockholm | Sweden |
| Orthopaedic and Trauma Center | Torino | Italy |
| Odense University Hospital | Idense | Denmark |
| Surgery and Perioperative Science, Umea University | Umea | Sweden |
| Ospedale Maggiore Policlinico | Milan | Italy |
| Hospital of Cruces | Bilbao | Spain |
| Niguarda Hospital | Milan | Italy |
| ASST di Monza | Monza | Italy |
| University Hospital of Aachen | Aachen | Germany |
| Cambridge University Hospital NHS Foundation Trust | Cambridge | United Kingdom |
| Intensive Care Unit, CHU Poiters | Poiters | France |
| Salford Royal Hospital NHS Foundation Trust | Salford | United Kingdom |
| Antwerp University Hospital and University of Antwerp | Edegem | Belgium |
| Maggiore Della Carità Hospital | Novara | Italy |
| University Hospitals Leuven | Leuven | Belgium |
| Clinical centre of Vojvodina, Faculty of Medicine, University of Novi Sad | Novi Sad | Serbia |
| CHR Citadelle | Liège | Belgium |
| University of Pécs | Pécs, | Hungary |
| Rigshospitale | Copenhagen | Denmark |
| Erasmus MC | Rotterdam | The Netherlands |
| Hadassah Hebrew University Medical Center | Jerusalem | Israel |
| CHU | Liège | Belgium |
| Hospital Universitario 12 de Octubre, | Madrid | Spain |
| University Medical Center Groningen, | Groningen | Netherlands |
| Sheffield Teaching Hospitals NHS Foundation Trust | Sheffield | United Kingdom |
| Lille University Hospital | Lille | France |
| Rambam Medical Center | Haifa | Israel |
| University Hospitals Southampton NHS Trust | Southampton | United Kingdom |
| Southmead Hospital | Bristol | United Kingdom |
| Bufalini Hospital | Cesena | Italy |
| University Hospital Heidelberg | Heidelberg | Germany |
| The Walton Center NHS Foundation Trust | Liverpool | United Kingdom |
| Emergency County Hospital | Timisoara | Romania |
| University Hospital of Grenoble | Grenoble | France |
| Azienda Ospedaliera Università di Padova | Padova | Italy |
| Leiden University Medical Center | Leiden | The Netherlands |
| Medical Center Haaglanden | The Hague | The Netherlands |
| Helsinki University Central Hospital | Helsinki | Finland |
| Turku University Hospital and University of Turku | Turku | Finland |
| Vall d'Hebron Research Institute, | Barcelona | Spain |
| Kaunas University of technology and Vilnius University | Vilnius | Lithuania |
| Neurosurgery, Rezekne Hospital | Rezekne | Latvia |
| NHS Lothian Health Board | Edinburgh | United Kingdom |
| Klinikum Ludwigsburg | Ludwigsburg | Germany |
| Elisabeth-TweeSteden Ziekenhuis | Tilburg | The Netherlands |
| St. Olavs Hospital, Trondheim University Hospital | Trondheim | Norway |
| Fondazione IRCCS Cà Granda Ospedale Maggiore Policlinico | Milano | Italy |
| Sophia Children’s Hospital | Rotterdam | The Netherlands |
| Kings college London | London | United Kingdom |
| Medical University | Vienna | Austria |
| University of Szeged | Szeged | Hungary |

Table: Hospitals recruiting to the CENTER TBI Registry
