# Supplementary material for: The burden of traumatic brain injury from low-energy falls among patients from 18 countries in the CENTER-TBI Registry: A comparative cohort study
Source: PLoS Med. 2021 Sep 14;18(9):e1003761. doi: 10.1371/journal.pmed.1003761 (PMC8509890; doi:10.1371/journal.pmed.1003761)
Supplement: S2 Table — All within-pathway low- versus high-energy differences in age, sex, pre-existing health, and anticoagulant/antiplatelet medication were significant (p < 0.001). *ED = discharged or died in emergency department. **ADM = admitted to hospital but did not receive critical care in study hospital. ***ICU = admitted to hospital and received critical care in study hospital. (DOCX) [file pmed.1003761.s010.docx]

|  | High Energy ED* | Low Energy ED* | High Energy ADM** | Low Energy ADM** | High Energy ICU*** | Low Energy ICU*** |
| --- | --- | --- | --- | --- | --- | --- |
| TOTAL(N) | **5510** | **3775** | **4195** | **3620** | **2310** | **633** |
| Median age (IQR) | 37(24-55) | 70(50-83) | 47(27-66) | 79(65-87) | 44(26-61) | 67(54-79) |
| Male | 3508(63·7) | 1729(45·8) | 2851(68·0) | 1811(50·0) | 1716(74·3) | 418(66·0) |
| Pre-injury ASA-PS classification |  |  |  |  |  |  |
| Normal healthy patient | 3361(61·0) | 835(22·1) | 2280(54·4) | 427(11·8) | 1161(50·3) | 87(13·7) |
| A patient with mild systemic disease | 1453(26·4) | 1364(36·1) | 1121(26·7) | 1203(33·2) | 596(25·8) | 189(29·9) |
| A patient with severe systemic disease | 481(8·7) | 1338(35·4) | 631(15·0) | 1723(47·6) | 340(14·7) | 271(42·8) |
| A patient with Life-threatening disease | 29(0·5) | 129(3·4) | 49(1·2) | 207(5·7) | 29(1·3) | 46(7·3) |
| Anticoagulants | 223(4·0) | 634(16·8) | 338(8·1) | 987(27·3) | 131(5·7) | 118(18·6) |
| Platelet Aggregate Inhibitors | 260(4·7) | 649(17·2) | 305(7·3) | 813(22·5) | 151(6·5) | 137(21·6) |

TABLE SHOWING: COMPARISON OF DEMOGRAPHIC AND COMORBID CHARACTERESTICS BY ENERGY TRANSFER AND CARE PATHWAY – All “within pathway” low v high energy differences in age, gender, pre-existing health and anticoagulant/antiplatelet medication were significant p<0.001. *ED= Discharged or died in Emergency Department, **ADM=Admitted to a hospital & not receiving critical care in study hospital, ***ICU = Admitted to hospital and received critical care in study hospital
