# Supplementary material for: The burden of traumatic brain injury from low-energy falls among patients from 18 countries in the CENTER-TBI Registry: A comparative cohort study
Source: PLoS Med. 2021 Sep 14;18(9):e1003761. doi: 10.1371/journal.pmed.1003761 (PMC8509890; doi:10.1371/journal.pmed.1003761)
Supplement: S3 Table — *Excluding 9,286 patients who were discharged from or died in ED and 722 with missing age/GCS sum score and/or extracranial injury details. AUC = 0.90. **AOR = 0.46 (95% CI 0.43 to 0.50) when same model omits age × energy transfer interaction—other variable AORs unchanged. CT, computed tomography; ED, emergency department; GCS, Glasgow Coma Score. (DOCX) [file pmed.1003761.s011.docx]

| Variable | Univariable Odds ratio of admission to ICU (95%CI) | Adjusted Odds ratio of admission to ICU (95%CI) |
| --- | --- | --- |
| Age (per year) | 0$\cdot$98 (0$\cdot$98- 0$\cdot$99) | 0$\cdot$99 (0$\cdot$99- $1\cdot$00) |
| Sex. Male  (Reference = Female) | $1\cdot76$ (1$\cdot62$- 1$\cdot$92) | 1$\cdot$24 (1$\cdot$09 - 1$\cdot$40) |
| Pre existing disease: Reference = None | | |
| Mild systemic disease  Severe systemic disease  Severe that is a constant threat to life  Not recorded | 0$\cdot73$ (0$\cdot66$ - 0$\cdot81$)  $0\cdot56$(0$\cdot51$ - 0$\cdot6$3)  $0\cdot54$ (0$\cdot42$ - 0$\cdot$70)  $1\cdot86$ (1$\cdot$56 - 2$\cdot23$) | 0$\cdot$96 (0$\cdot$82 - 1$\cdot$13)  1$\cdot$19 (1$\cdot$00 - 1$\cdot$43)  1$\cdot$24 (0$\cdot$85 - 1$\cdot$78)  0$\cdot$75 (0$\cdot$55 - 0$\cdot$98) |
| Marshall Classification: Reference = Gd I no visible pathology | | |
| II CT abnormality Cisterns present (Midline Shift 0-5mm)  III Diffuse Injury (Cisterns compressed/ absent with midline shift 0-5mm)  IV Diffuse Injury (Midline Shift > 5mm)  V Surgically-evacuated mass lesion  VI Non-evacuated mass lesion | $5\cdot13$ (4$\cdot65$ - 5$\cdot66$)  $44\cdot61$(29$\cdot30$- 67$\cdot$92)  $12\cdot88$ (9$\cdot20$ - 18$\cdot04$)  $36\cdot73$ (29$\cdot56$ - 45$\cdot64$)  $18\cdot55$ (15$\cdot28$ - 22$\cdot52$) | 3$\cdot$35 ($2\cdot$96 - 3$\cdot$79)  9$\cdot$76 (5$\cdot$69- 17$\cdot$09)  6$\cdot$73 (4$\cdot$21 - 10$\cdot$75)  24$\cdot$04 (18$\cdot$54 - 31$\cdot$35)  6$\cdot$72 (5$\cdot$13 - 8$\cdot$79) |
| GCS in ED | 0$\cdot65$ (0$\cdot63$ - 0$\cdot66$) | 0$\cdot$73 (0$\cdot$72 - 0$\cdot$75) |
| ED Pupillary reactivity: Reference = Bilaterally reactive | | |
| One pupil unreactive  Two pupils unreactive  Not recorded | $5\cdot48$ (4$\cdot40$ - 6$\cdot83$)  $30\cdot84$ (21$\cdot90$ - 0$\cdot$87)  $7\cdot$84(5$\cdot34$ - 11$\cdot5$) | 1$\cdot$39 (0$\cdot$97 - 1$\cdot$98)  0$\cdot$54 (0$\cdot$33 - 0$\cdot$87)  0$\cdot$83 (0$\cdot$46 - 1$\cdot$53) |
| Significant Extracranial Injury (AIS 3+)  (Reference = No significant Extracranial Injury (AIS <3)) | $2\cdot04$ (1$\cdot86$ - 2$\cdot23$) | 1$\cdot$60 (1$\cdot$40 - 1$\cdot$82) |
| Arriving at ED intubated  (Reference = Arriving at ED not intubated) | 119.4 (89$\cdot$48 - 159$\cdot$21) | 4$\cdot$96 (3$\cdot$47 - 7$\cdot$18) |
| Low Energy Transfer  ((Reference = High Energy Transfer) | 0$\cdot3$6 (0$\cdot33$ – 0$\cdot$39) | 0$\cdot$77 (0$\cdot$53 – $1\cdot$12)****** |
| Age: Energy Transfer (Low Energy) Interaction | 0$\cdot$99 (0$\cdot$99 - 1$\cdot$00) | 0$\cdot$99 (0$\cdot$99 - 1$\cdot$00) |

TABLE SHOWING : MULTIVARIABLE ANALYSIS OF FACTORS (age, sex, pre-existing disease status, Marshall CT brain injury classification, abnormality, ED GCS and pupillary reactivity, presence of significant extracranial injury, being intubated on ED arrival, causal energy transfer mechanism and its interaction with age) PREDICTING ICU ADMISSION IN 11673* PATIENTS FROM THE CENTER TBI REGISTRY *excluding 9286 patients discharged from/ dying in ED and 722 with missing Age/GCS Sum Score +/or extracranial injury details. AUC = 0.90. ****AOR = 0·46 (95% CI 0·43-0·50**) when same model omits age energy transfer interaction – other variable AORs unchanged ( GCS=Glasgow Coma Score, ER= Emergency Department, CT= CT brain scan, CI=Confidence Interval, IQR=Interquartile range, AIS=Abbreviated Injury Scale,
