# Supplementary material for: The burden of traumatic brain injury from low-energy falls among patients from 18 countries in the CENTER-TBI Registry: A comparative cohort study
Source: PLoS Med. 2021 Sep 14;18(9):e1003761. doi: 10.1371/journal.pmed.1003761 (PMC8509890; doi:10.1371/journal.pmed.1003761)
Supplement: S4 Table — *Excluding patients who died in the ED (n = 69), arrived as secondary transfers (n = 2,706), or had missing age/CT abnormality/GCS sum score/extracranial injury (n = 873). GCS considered as binary category in accordance with guidance for hospital admission [8]. Area under receiver operating characteristic curve (AUC) = 0.81. AIS, Abbreviated Injury Scale; CT, computed tomography; ED, emergency department; GCS, Glasgow Coma Score. (DOCX) [file pmed.1003761.s012.docx]

| Variable | Univariable Odds ratio of admission to hospital (95%CI) | Adjusted Odds ratio of admission to hospital (95%CI) |
| --- | --- | --- |
| Age (per year) | 1$\cdot$01 (1$\cdot$01-1$\cdot$01) | 1$\cdot$01 (1$\cdot$01-1$\cdot$01) |
| Sex. Male  (Reference = Female) | 1∙28 (1∙21-1∙36) | 1∙16 (1∙08-1∙25) |
| Pre existing disease: Reference = None | | |
| Mild systemic disease  Severe systemic disease  Severe that is a constant threat to life  Not recorded | 1∙14 (1∙06-1$\cdot22$)  1$\cdot77$ (1$\cdot64$-1$\cdot91$)  2∙34 (1$\cdot91$-2$\cdot86$)  1$\cdot$39 (1$\cdot$17-1$\cdot$66) | 0∙96 (0∙87-1$\cdot$05)  1$\cdot$47 (1$\cdot$32-1$\cdot$65)  2∙04 (1$\cdot$60-2$\cdot$60)  0$\cdot$62 (0$\cdot$49-0$\cdot$78) |
| Presence of CT brain scan Abnormality  (Reference= No injury on CT brain scan) | 16$\cdot$56 (14$\cdot79$-18$\cdot54$) | 9$\cdot$70 (8$\cdot$63-10$\cdot$95) |
| GCS 15 in ED**  (Reference = GCS <15 in ED) | 0$\cdot20$ (0$\cdot18$-0$\cdot21$) | 0$\cdot$35 (0$\cdot$32-0$\cdot$38) |
| Significant Extracranial Injury (AIS 3+)  (Reference = No significant Extracranial Injury (AIS <3)) | 2$\cdot93$ (2$\cdot76$-3$\cdot12$) | 2$\cdot$40 (2$\cdot$24-2$\cdot$58) |
| Arriving at ED intubated  (Reference = Arriving at ED not intubated) | $226\cdot01$(93$\cdot83$-544$\cdot35$) | 41$\cdot$11 (1$8\cdot$84-11$5\cdot$65) |
| Low Energy Transfer  (Reference = High Energy Transfer) | 0$\cdot95$(0$\cdot89$-1$\cdot00$) | 0$\cdot$82 (0$\cdot$34-0$\cdot$53) |
| Age: Energy Transfer (Low Energy) Interaction | 1$\cdot$01 (1$\cdot$01-1$\cdot$01) | 1$\cdot$01 (1$\cdot$01-1$\cdot$01) |

TABLE SHOWING : MULTIVARIABLE ANALYSIS OF FACTORS (age, sex, pre-existing disease status, presence of CT brain abnormality, ED GCS < 15, presence of significant extracranial injury, being intubated on ED arrival, causal energy transfer mechanism and its interaction with age) PREDICTING HOSPITAL ADMISSION IN 18035* PATIENTS FROM THE CENTER TBI REGISTRY *excluding patients who died in ER n=69, arrived as secondary transfers n= 2706, or with missing Age / CT Abnormality / GCS sum score / extracranial injury n=873. GCS Considered as binary category in accordance with guidance for hospital admission (8). Area Under Receiver Operator Characteristic Curve (AUC) = 0.81 . GCS=Glasgow Coma Score, ED= Emergency Department, CT= CT brain scan, CI=Confidence Interval, IQR=Interquartile range, AIS=Abbreviated Injury Scale,
