# Supplementary material for: The burden of traumatic brain injury from low-energy falls among patients from 18 countries in the CENTER-TBI Registry: A comparative cohort study
Source: PLoS Med. 2021 Sep 14;18(9):e1003761. doi: 10.1371/journal.pmed.1003761 (PMC8509890; doi:10.1371/journal.pmed.1003761)
Supplement: S6 Table — *Excluding 432 patients with missing GCS sum score/age and/or discharge status. Area under receiver operating characteristic curve (AUC) = 0.86. AIS, Abbreviated Injury Scale; CT, computed tomography; ED, emergency department; GCS, Glasgow Coma Score. (DOCX) [file pmed.1003761.s014.docx]

| Variable | Univariable Odds ratio of hospital mortality (95%CI) | Adjusted Odds ratio (95%CI) of hospital mortality |
| --- | --- | --- |
| Age (per year) | 1.02 (1.02-1.03) | 1.02 (1.01 - 1.03) |
| Sex. Male (Reference = Female) | 0.85 (0.71- 1.02) | 0.44 (0.24 - 0.82) |
| Pre existing disease status: Reference = None |  |  |
| Mild systemic disease  Severe systemic disease  Severe systemic disease that is a constant threat to life  Not recorded | 1.57 (1.26- 1.96)  2.54 (2.04- 3.16)  4.93 (3.08- 7.89)  2.35 (1.77- 3.11) | 0.92 (0.69 - 1.24)  1.45 (1.05 - 2.02)  3.32 (1.79 - 6.07)  1.35 (0.81 - 2.26) |
| Pre injury Anticoagulation status: Reference = not taking |  | |
| Taking anticoagulants  Not recorded | 2.51 (1.95- 3.23)  1.99 (1.58- 2.49) | 1.35 (0.97 - 1.89)  1.43 (0.94 - 2.16) |
| Marshall Classification: Reference = Gd I (no visible pathology) |  | |
| II CT abnormality Cisterns present (Midline Shift 0-5mm)  III Diffuse Injury (Cisterns compressed/ absent with midline shift 0-5mm)  IV Diffuse Injury (Midline Shift > 5mm)  V Surgically-evacuated mass lesion  VI Non-evacuated mass lesion > 25cm | 1.21 (0.89- 1.65)  13.11(8.67-19.82)  8.28 (5.21- 13.17)  2.77 (1.98- 3.88)  11.85(8.53-16.47) | 0.77 (0.54 - 1.09)  5.49 (3.41 - 8.91)  3.70 (2.19 - 6.27)  1.21 (0.82 - 1.79)  4.32 (2.95 - 6.38) |
| ED GCS | 0.87 (0.85 - 0.88) | 0.91 (0.89 - 0.93) |
| ED Pupillary Reactivity: Reference = Bilaterally reactive |  | |
| One pupil unreactive  Two pupils unreactive  Not recorded | 3.81 (2.86- 5.07)  13.27(10.57-16.68)  1.90 (1.14 - 3.05 | 2.20 (1.57 - 3.08)  7.37 (5.57 - 9.80)  1.90 (1.15 - 3.06) |
| Significant Extracranial injury (AIS 3+)  (Reference = No significant Extracranial Injury (AIS <3)) | 1.65 (1.36 - 2.01) | 1.31 (1.02 - 1.70) |
| Low Energy Transfer  (Reference = High Energy Transfer) | 1.51 (1.26 - 1.81) | 0.42 (0.16 - 1.11) |
| Age: Sex (Male) Interaction | 1.02 (1.02 - 1.03) | 1.01(1.00 - 1.02) |
| Age: Energy Mechanism(Low Energy) Interaction | 1.02 (1.02 - 1.03) | 1.01 (1.00 - 1.03) |

**TABLE SHOWING : MULTIVARIABLE ANALYSIS OF FACTORS (age, sex and their interaction, pre-existing disease status, pre injury anticoagulation status, Marshall CT brain injury classification, ED GCS and pupillary reactivity, presence of significant extracranial injury, causal energy transfer mechanism and its interaction with age) PREDICTING IN HOSPITAL MORTALITY IN 3739* ICU ADMISSION PATIENTS FROM THE CENTER TBI REGISTRY *excluding 432 patients with missing GCS Sum Score/Age +/or discharge status AUC=0.86 GCS=Glasgow Coma Score, ED= Emergency Department, CT= CT brain scan, CI=Confidence Interval, IQR=Interquartile range, AIS=Abbreviated Injury Scale,**
