# Supplementary material for: The burden of traumatic brain injury from low-energy falls among patients from 18 countries in the CENTER-TBI Registry: A comparative cohort study
Source: PLoS Med. 2021 Sep 14;18(9):e1003761. doi: 10.1371/journal.pmed.1003761 (PMC8509890; doi:10.1371/journal.pmed.1003761)
Supplement: S7 Table — GCS, Glasgow Coma Score. *ED = discharged from or died in the emergency department. **ADM = admitted to hospital but did not receive critical care in study hospital. ***ICU = admitted to hospital and received critical care in study hospital. (DOCX) [file pmed.1003761.s015.docx]

|  | **Low Energy TBI (N, %)** | **Unknown Energy level (N,%)** | **High Energy TBI (N, %)** |
| --- | --- | --- | --- |
| TOTAL MECHANISM OF INJURY(N) | **8622** | **1101** | **13059** |
| ***Demographic characteristics***  Median age (IQR) | 74(56-84) | 60 (40-77) | 42(25-60) |
| - Under 16 years | 91(1·1) | 10 (0·9) | 488 (3·7) |
| - 16-64 years | 2848(33·0) | 619 (56·2) | 9934(76·1) |
| - 65 years and over | 5682 (65·9) | 472 (42·9) | 2635(20·2) |
| ***Male*** |  |  |  |
| 1. Overall | 4353(50·5) | 639 (58·0) | 8833(67·6) |
| 1. under 16 | 55(60·4) | 4 (40·0) | 316(64·8) |
| 1. 16-64 | 1787(62·7) | 416 (67·2) | 7044(70·9) |
| (iv) 65 and over | 2510(44·2) | 219 (46·4) | 1472(55·9) |
| *Place of injury* |  |  |  |
| Street/highway | 996(11·6) | 110(10·0) | 6328(48·5) |
| Home/domestic | 5435(63·0) | 379(34·4) | 2860(21·9) |
| Work/school | 159(1·8) | 69(6·3) | 587(4·5) |
| Sport/Recreational | 50(0·6) | - | 671(5·1) |
| Public location | 1394(16·2) | 153(13·9) | 2000(15·3) |
| Other | 427(5·0) | 137(12·4) | 308(2·4) |
| Unknown | 156(1·8) | 210(19·1) | 292(2·2) |
| Missing | 5(0·1) | 43(3·9) | 13(0·1) |
| ***Care Pathway*** |  |  |  |
| ED* | 3775(43·8) | 553 (50·2) | 5511(42·2) |
| Admission ** | 3859(44·8) | 347 (31·5) | 4365(33·4) |
| ICU*** | 988(11·5) | 201 (18·3) | 3183(24·4) |
| ***Pre-injury health status and medical history*** |  |  |  |
| Pre-injury ASA-PS classification |  |  |  |
| - Normal healthy patient | 1403(16·3) | 382 (34·7) | 7285(55·8) |
| - A patient with mild systemic disease | 2875(33·3) | 278 (25·2) | 3391(26·0) |
| - A patient with severe systemic disease | 3536(41·0) | 228 (20·7) | 1569(12·0) |
| - A patient with Life-threatening disease | 407(4·7) | 12 (1·1) | 116(0·9) |
| Anticoagulants | 1834(21·3) | 160 (14·5) | 744(5·7) |
| Platelet Aggregate Inhibitors | 1683(19.5) | 108 (9·8) | 783(6·0) |
| Both Anticoagulants and Platelet Aggregate Inhibitors | 250(2·9) | 17 (1·5) | 120(0·9) |
| Patients with intracranial lesions taking anticoagulants | 373(20·9) | 25(11·6) | 207(6·5) |
| Patients with intracranial lesions taking Platelet Aggregate Inhibitors | 410(23·0) | 18 (8·3) | 269(8·5) |
| Patients with intracranial lesions taking both anticoagulants and Platelet Aggregate Inhibitors | 77(4·3) | 5 (2·3) | 37(1·2) |
| **PATHOPHYSIOLOGY AT ED Arrival** |  |  |  |
| GCS AT ED ARRIVAL (median(IQR)) * | 15(14-15) | 15(14-15) | 15(14-15) |
| Mild ( GCS 13-15) | 7432(86·2) | 775 (70·4) | 10270(78·6) |
| Moderate (GCS 9-12) | 350(4·1) | 58 (5·3) | 480(3·7) |
| Severe(GCS 3-8) | 316(3·7) | 66 (6·0) | 879(6·7) |
| No Sum | 469 (5·4) | 111 (10·1) | 1366(10·5) |
| Hypoxia | 104 (1·2) | 11 (1·0) | 183(1·4) |
| Hypotension | 112(1·3) | 11 (1·0) | 322(2·5) |
| ***Pupillary reactivity*** |  |  |  |
| None Reacting | 133(1·5) | 24 (2·2) | 419(3·2) |
| One reacting | 170(2·0) | 27 (2·5) | 313(2·4) |
| Both reacting | 7715 (89·5) | 892 (81·0) | 11694 (89·5) |
| *Referral* |  |  |  |
| Secondary referral – Arrived from another hospital | 972(11·3) | 234 (21·3) | 1720(13·2) |

SUPPLEMENTAL TABLE: DEMOGRAPHICS, INJURY MECHANISM, CO-MORBIDITY PRESENTING PHYSIOLOGY AND CARE PATHWAY-COMPARATIVE “ANALYSIS” OF THE CENTER-TBI REGISTRY HIGH, LOW AND UNKNOWN ENERGY TRANSFER COHORTS GCS=Glasgow Coma Score.. *ED= Discharged or died in Emergency Department, **ADM=Admitted to a hospital & not receiving critical care in study hospital, ***ICU = Admitted to hospital and received critical care in study hospital
