# Supplementary material for: The burden of traumatic brain injury from low-energy falls among patients from 18 countries in the CENTER-TBI Registry: A comparative cohort study
Source: PLoS Med. 2021 Sep 14;18(9):e1003761. doi: 10.1371/journal.pmed.1003761 (PMC8509890; doi:10.1371/journal.pmed.1003761)
Supplement: S8 Table — AIS, Abbreviated Injury Scale; ASH, acute subdural haematoma; CT, computed tomography; EDH, extradural haematoma; ICP, intracranial pressure; ISS, Injury Severity Score. **Denominator is those with abnormal CT brain scan. (DOCX) [file pmed.1003761.s016.docx]

|  | Low Energy TBI (N, %) | Unknown Energy Mechanism(N,%) | High Energy TBI (N, %) |
| --- | --- | --- | --- |
| TOTAL MECHANISM OF INJURY(N) | **8622** | **1101** | **13059** |
| *CT characteristics* |  |  |  |
| %(95%CI) Abnormal CT | 2520(29·2) | 293 (26∙6) | 4226(32·4) |
| %(95% CI) of Abnormal CT = Intracranial lesion ** | 1782(70·7) | 216 (73∙7) | 3177(75·2) |
| EDH small ** | 163 (6·5) | 25 (8∙5) | 456(10·8) |
| EDH large* * | 64(2·5) | 15 (5∙1) | 175 (4·1) |
| ASDH small** | 765(30·4) | 90 (30∙7) | 1271(30·1) |
| ASDH large** | 463(18·4) | 61 (20∙8) | 520(12·3) |
| Contusions small ** | 751(29·8) | 89 (30∙4) | 1698(40·2) |
| Contusions large** | 163(6·5) | 35 (11∙9) | 404(9·6) |
| Compressed basal cisterns** | 243(9∙6) | 38 (13∙0) | 548(13·0) |
| Midline Shift** | 709(28·2) | 97 (33·1) | 883(20·9) |
| Sub-Arachnoid Haemorrhage** | 1112(44·1) | 131 (44·7 ) | 2397(56·7) |
|  |  |  |  |
| *Marshall Classificaton of CT findings* |  |  |  |
| II: Cisterns present with Midline Shift 0-5mm and / or lesions present (high/mixed density < 25cm) may include bone fragments | 1761(20·5) | 190(18·2) | 3141(24·3) |
| III Diffuse Injury III Cisterns compressed/ absent with midline shift 0-5mm(high/mixed density < 25cm) | 36(0·4) | 6(0·6) | 181(1·4) |
| IV Diffuse Injury (Midline Shift > 5mm) (high/mixed density < 25cm) | 94(1·1) | 9(0·9) | 107(0·8) |
| V Surgically-evacuated mass lesion | 363(4·2) | 62(5·9) | 396(3·1) |
| VI Non-evacuated mass lesion >25cm | 266(3·1) | 16(1·5) | 401(3.1) |
| Median Head-Neck AIS (IQR) | 1(1-2) | 1(1-2) | 2(1-3) |
| Median Cervical Spine AIS(IQR) | 0(0-0) | 0(0-0) | 0(0-0) |
| Median ISS (IQR) | 6(3-12) | 5(2-12) | 9(4-20) |
| Median Extracranial ISS (IQR) | 2(0-8) | 1(0-5) | 5(1-13) |
| % (95%CI) Arrived Intubated | 316(3·7) | 6·1(67) | 1496(11·5) |
| Key Interventions |  |  |  |
| % (95%CI) Had at least one Key emergency Intervention | 645(7·5) | 98 (8·9) | 1752(13·4) |
| % (95%CI) Key emergency Interventions performed |  |  |  |
| Craniotomy | 366(4·2) | 63(5·7) | 401(3·1) |
| ICP insertion | 144(1·7) | 32 (2·9) | 609(4·7) |
| Decompressive Craniectomy | 74 (0·9) | 6 (0·5) | 201(1·5) |
| External Fixation Limb | 17(0·2) | 1 (0·1) | 276(2·1) |
| ^Others | 76(0·9) | 10 (0·9) | 369(2·8) |
| %(95%CI) survival to hospital discharge(alive) -destinations shown below | 8082(93∙7) | 1011 (91∙8) | 12146(93·0) |
| Discharged home | 5866(68·0) | 628 (57·0) | 9458(72·4) |
| Discharged other hospital | 800(9·3) | 168 (15·3) | 1351(10·3) |
| Discharged to rehabilitation | 445(5·2) | 40 (3·6) | 776(5·9) |
| Discharged to nursing home | 833(9·7) | 86 (7·8) | 289(2·2) |

SUPPLEMENTAL TABLE : IMAGING FINDINGS, INJURY SEVERITY, THERAPEUTIC INTERVENTIONS, DISCHARGE STATUS - COMPARATIVE ANALYSIS” OF THE CENTER-TBI REGISTRY HIGH, LOW AND UNKNOWN ENERGY TRANSFER COHORTS. “” Denominator = those with abnormal CT brain scan. CT= CT brain scan, CI=Confidence Interval, IQR=Interquartile range, AIS=Abbreviated Injury Scale, ISS = Injury Severity Score, EDH = Extradural Haematoma, ASH=Acute Subdural Haematoma, ICP=Intracranial Pressure
