# Supplementary material for: The burden of traumatic brain injury from low-energy falls among patients from 18 countries in the CENTER-TBI Registry: A comparative cohort study
Source: PLoS Med. 2021 Sep 14;18(9):e1003761. doi: 10.1371/journal.pmed.1003761 (PMC8509890; doi:10.1371/journal.pmed.1003761)
Supplement: S1 Text — (DOCX) [file pmed.1003761.s017.docx]

**Institutional affiliations of CENTER TBI participants and investigators**

^1^ Department of Physiology and Pharmacology, Section of Perioperative Medicine and Intensive Care, Karolinska Institutet, Stockholm, Sweden

^2^ János Szentágothai Research Centre, University of Pécs, Pécs, Hungary

^3^ Division of Surgery and Clinical Neuroscience, Department of Physical Medicine and Rehabilitation, Oslo University Hospital and University of Oslo, Oslo, Norway

^4^ Department of Neurosurgery, University Hospital Northern Norway, Tromso, Norway

^5^ Department of Physical Medicine and Rehabilitation, University Hospital Northern Norway, Tromso, Norway

^6^ Trauma Surgery, Medical University Vienna, Vienna, Austria

^7^ Department of Anesthesiology & Intensive Care, University Hospital Nancy, Nancy, France

^8^ Raymond Poincare hospital, Assistance Publique – Hopitaux de Paris, Paris, France

^9^ Department of Anesthesiology & Intensive Care, S Raffaele University Hospital, Milan, Italy

^10^ Department of Neurosurgery, Radboud University Medical Center, Nijmegen, The Netherlands

^11^ Department of Neurosurgery, University of Szeged, Szeged, Hungary

^12^ International Projects Management, ARTTIC, Munchen, Germany

^13^ Department of Neurology, Neurological Intensive Care Unit, Medical University of Innsbruck, Innsbruck, Austria

^14^ Department of Neurosurgery & Anesthesia & intensive care medicine, Karolinska University Hospital, Stockholm, Sweden

^15^ NIHR Surgical Reconstruction and Microbiology Research Centre, Birmingham, UK

^16^ Anesthesie-Réanimation, Assistance Publique – Hopitaux de Paris, Paris, France

^17^ Department of Anesthesia & ICU, AOU Città della Salute e della Scienza di Torino - Orthopedic and Trauma Center, Torino, Italy

^18^ Department of Neurology, Odense University Hospital, Odense, Denmark

^19^ BehaviourWorks Australia, Monash Sustainability Institute, Monash University, Victoria, Australia

^20^ Department of Public Health, Faculty of Health Sciences and Social Work, Trnava University, Trnava, Slovakia

^21^ Quesgen Systems Inc., Burlingame, California, USA

^22^ Australian & New Zealand Intensive Care Research Centre, Department of Epidemiology and Preventive Medicine, School of Public Health and Preventive Medicine, Monash University, Melbourne, Australia

^23^ Department of Surgery and Perioperative Science, Umeå University, Umeå, Sweden

^24^ Department of Neurosurgery, Medical School, University of Pécs, Hungary and Neurotrauma Research Group, János Szentágothai Research Centre, University of Pécs, Hungary

^25^ Department of Medical Psychology, Universitätsklinikum Hamburg-Eppendorf, Hamburg, Germany

^26^ Brain Physics Lab, Division of Neurosurgery, Dept of Clinical Neurosciences, University of Cambridge, Addenbrooke’s Hospital, Cambridge, UK

^27^ Neuro ICU, Fondazione IRCCS Cà Granda Ospedale Maggiore Policlinico, Milan, Italy

^28^ ANZIC Research Centre, Monash University, Department of Epidemiology and Preventive Medicine, Melbourne, Victoria, Australia

^29^ Department of Neurosurgery, Hospital of Cruces, Bilbao, Spain

^30^ NeuroIntensive Care, Niguarda Hospital, Milan, Italy

^31^ School of Medicine and Surgery, Università Milano Bicocca, Milano, Italy

^32^ NeuroIntensive Care, ASST di Monza, Monza, Italy

^33^Department of Neurosurgery, Medical Faculty RWTH Aachen University, Aachen, Germany

^34^ Department of Anesthesiology and Intensive Care Medicine, University Hospital Bonn, Bonn, Germany

^35^ Department of Anesthesia & Neurointensive Care, Cambridge University Hospital NHS Foundation Trust, Cambridge, UK

^36^ School of Public Health & PM, Monash University and The Alfred Hospital, Melbourne, Victoria, Australia

^37^ Radiology/MRI department, MRC Cognition and Brain Sciences Unit, Cambridge, UK

^38^ Institute of Medical Psychology and Medical Sociology, Universitätsmedizin Göttingen, Göttingen, Germany

^39^ Oxford University Hospitals NHS Trust, Oxford, UK

^40^ Intensive Care Unit, CHU Poitiers, Potiers, France

^41^ University of Manchester NIHR Biomedical Research Centre, Critical Care Directorate,  Salford Royal Hospital NHS Foundation Trust, Salford, UK

^42^ Movement Science Group, Faculty of Health and Life Sciences, Oxford Brookes University, Oxford, UK

^43^ Department of Neurosurgery, Antwerp University Hospital and University of Antwerp, Edegem, Belgium

^44^ Department of Anesthesia & Intensive Care, Maggiore Della Carità Hospital, Novara, Italy

^45^ Department of Neurosurgery, University Hospitals Leuven, Leuven, Belgium

^46^ Department of Neurosurgery, Clinical centre of Vojvodina, Faculty of Medicine, University of Novi Sad, Novi Sad, Serbia

^47^ Division of Anaesthesia, University of Cambridge, Addenbrooke’s Hospital, Cambridge, UK

^48^ Center for Stroke Research Berlin, Charité – Universitätsmedizin Berlin, corporate member of Freie Universität Berlin, Humboldt-Universität zu Berlin, and Berlin Institute of Health, Berlin, Germany

^49^ Intensive Care Unit, CHR Citadelle, Liège, Belgium

^50^ Department of Anaesthesiology and Intensive Therapy, University of Pécs, Pécs, Hungary

^51^ Departments of Neurology, Clinical Neurophysiology and Neuroanesthesiology, Region Hovedstaden Rigshospitalet, Copenhagen, Denmark

^52^ National Institute for Stroke and Applied Neurosciences, Faculty of Health and Environmental Studies, Auckland University of Technology, Auckland, New Zealand

^53^ Department of Neurology, Erasmus MC, Rotterdam, the Netherlands

^54^ Department of Anesthesiology and Intensive care, University Hospital Northern Norway, Tromso, Norway

^55^ Department of Neurosurgery, Hadassah-hebrew University Medical center, Jerusalem, Israel

^56^ Fundación Instituto Valenciano de Neurorrehabilitación (FIVAN), Valencia, Spain

^57^ Department of Neurosurgery, Shanghai Renji hospital, Shanghai Jiaotong University/school of medicine, Shanghai, China

^58^ Karolinska Institutet, INCF International Neuroinformatics Coordinating Facility, Stockholm, Sweden

^59^ Emergency Department, CHU, Liège, Belgium

^60^ Neurosurgery clinic, Pauls Stradins Clinical University Hospital, Riga, Latvia

^61^ Department of Computing, Imperial College London, London, UK

^62^ Department of Neurosurgery, Hospital Universitario 12 de Octubre, Madrid, Spain

^63^ Department of Anesthesia, Critical Care and Pain Medicine, Medical University of Vienna, Austria

^64^ Department of Public Health, Erasmus Medical Center-University Medical Center, Rotterdam, The Netherlands

^65^ College of Health and Medicine, Australian National University, Canberra, Australia

^66^ Department of Neurosurgery, Neurosciences Centre & JPN Apex trauma centre, All India Institute of Medical Sciences, New Delhi-110029, India

^67^ Department of Neurosurgery, Erasmus MC, Rotterdam, the Netherlands

^68^ Department of Neurosurgery, Oslo University Hospital, Oslo, Norway

^69^ Division of Psychology, University of Stirling, Stirling, UK

^70^ Division of Neurosurgery, Department of Clinical Neurosciences, Addenbrooke’s Hospital & University of Cambridge, Cambridge, UK

^71^ Department of Neurology, University of Groningen, University Medical Center Groningen, Groningen, Netherlands

^72^ Neurointensive Care , Sheffield Teaching Hospitals NHS Foundation Trust, Sheffield, UK

^73^ Salford Royal Hospital NHS Foundation Trust Acute Research Delivery Team, Salford, UK

^74^ Department of Intensive Care and Department of Ethics and Philosophy of Medicine, Erasmus Medical Center, Rotterdam, The Netherlands

^75^ Department of Clinical Neuroscience, Neurosurgery, Umeå University, Umeå, Sweden

^76^ Hungarian Brain Research Program - Grant No. KTIA_13_NAP-A-II/8, University of Pécs, Pécs, Hungary

^77^ Department of Anaesthesiology, University Hospital of Aachen, Aachen, Germany

^78^ Cyclotron Research Center , University of Liège, Liège, Belgium

^79^ Centre for Urgent and Emergency Care Research (CURE), Health Services Research Section, School of Health and Related Research (ScHARR), University of Sheffield, Sheffield, UK

^80^ Emergency Department, Salford Royal Hospital, Salford UK

^81^ Institute of Research in Operative Medicine (IFOM), Witten/Herdecke University, Cologne, Germany

^82^ VP Global Project Management CNS, ICON, Paris, France

^83^ Department of Anesthesiology-Intensive Care, Lille University Hospital, Lille, France

^84^ Department of Neurosurgery, Rambam Medical Center, Haifa, Israel

^85^ Department of Anesthesiology & Intensive Care, University Hospitals Southhampton NHS Trust, Southhampton, UK

^86^ Cologne-Merheim Medical Center (CMMC), Department of Traumatology, Orthopedic Surgery and Sportmedicine, Witten/Herdecke University, Cologne, Germany

^87^ Intensive Care Unit, Southmead Hospital, Bristol, Bristol, UK

^88^ Department of Neurological Surgery, University of California, San Francisco, California, USA

^89^ Department of Anesthesia & Intensive Care,M. Bufalini Hospital, Cesena, Italy

^90^ Department of Neurosurgery, University Hospital Heidelberg, Heidelberg, Germany

^91^ Department of Neurosurgery, The Walton centre NHS Foundation Trust, Liverpool, UK

^92^ Department of Medical Genetics, University of Pécs, Pécs, Hungary

^93^ Department of Neurosurgery, Emergency County Hospital Timisoara , Timisoara, Romania

^94^ School of Medical Sciences, Örebro University, Örebro, Sweden

^95^ Institute for Molecular Medicine Finland, University of Helsinki, Helsinki, Finland

^96^ Analytic and Translational Genetics Unit, Department of Medicine; Psychiatric & Neurodevelopmental Genetics Unit, Department of Psychiatry; Department of Neurology, Massachusetts General Hospital, Boston, MA, USA

^97^ Program in Medical and Population Genetics; The Stanley Center for Psychiatric Research, The Broad Institute of MIT and Harvard, Cambridge, MA, USA

^98^ Department of Radiology, University of Antwerp, Edegem, Belgium

^99^ Department of Anesthesiology & Intensive Care, University Hospital of Grenoble, Grenoble, France

^100^ Department of Anesthesia & Intensive Care, Azienda Ospedaliera Università di Padova, Padova, Italy

^101^ Dept. of Neurosurgery, Leiden University Medical Center, Leiden, The Netherlands and Dept. of Neurosurgery, Medical Center Haaglanden, The Hague, The Netherlands

^102^ Department of Neurosurgery, Helsinki University Central Hospital

^103^ Division of Clinical Neurosciences, Department of Neurosurgery and Turku Brain Injury Centre, Turku University Hospital and University of Turku, Turku, Finland

^104^ Department of Anesthesiology and Critical Care, Pitié -Salpêtrière Teaching Hospital, Assistance Publique, Hôpitaux de Paris and University Pierre et Marie Curie, Paris, France

^105^ Neurotraumatology and Neurosurgery Research Unit (UNINN), Vall d'Hebron Research Institute, Barcelona, Spain

^106^ Department of Neurosurgery, Kaunas University of technology and Vilnius University, Vilnius, Lithuania

^107^ Department of Neurosurgery, Rezekne Hospital, Latvia

^108^ Department of Anaesthesia, Critical Care & Pain Medicine NHS Lothian & University of Edinburg, Edinburgh, UK

^109^ Director, MRC Biostatistics Unit, Cambridge Institute of Public Health, Cambridge, UK

^110^ Department of Physical Medicine and Rehabilitation, Oslo University Hospital/University of Oslo, Oslo, Norway

^111^ Division of Orthopedics, Oslo University Hospital, Oslo, Norway

^112^ Institue of Clinical Medicine, Faculty of Medicine, University of Oslo, Oslo, Norway

^113^ Broad Institute, Cambridge MA Harvard Medical School, Boston MA, Massachusetts General Hospital, Boston MA, USA

^114^ National Trauma Research Institute, The Alfred Hospital, Monash University, Melbourne, Victoria, Australia

^115^ Department of Neurosurgery, Odense University Hospital, Odense, Denmark

^116^ International Neurotrauma Research Organisation, Vienna, Austria

^117^ Klinik für Neurochirurgie, Klinikum Ludwigsburg, Ludwigsburg, Germany

^118^ Division of Biostatistics and Epidemiology, Department of Preventive Medicine, University of Debrecen, Debrecen, Hungary

^119^ Department Health and Prevention, University Greifswald, Greifswald, Germany

^120^ Department of Anaesthesiology and Intensive Care, AUVA Trauma Hospital, Salzburg, Austria

^121^ Department of Neurology, Elisabeth-TweeSteden Ziekenhuis, Tilburg, the Netherlands

^122^ Department of Neuroanesthesia and Neurointensive Care, Odense University Hospital, Odense, Denmark

^123^ Department of Neuromedicine and Movement Science, Norwegian University of Science and Technology, NTNU, Trondheim, Norway

^124^ Department of Physical Medicine and Rehabilitation, St.Olavs Hospital, Trondheim University Hospital, Trondheim, Norway

^125^ Department of Neurosurgery, University of Pécs, Pécs, Hungary

^126^ Division of Neuroscience Critical Care, John Hopkins University School of Medicine, Baltimore, USA

^127^ Department of Neuropathology, Queen Elizabeth University Hospital and University of Glasgow, Glasgow, UK

^128^ Dept. of Department of Biomedical Data Sciences, Leiden University Medical Center, Leiden, The Netherlands

^129^ Department of Pathophysiology and Transplantation, Milan University, and Neuroscience ICU, Fondazione IRCCS Cà Granda Ospedale Maggiore Policlinico, Milano, Italy

^130^ Department of Radiation Sciences, Biomedical Engineering, Umeå University, Umeå, Sweden

^131^ Perioperative Services, Intensive Care Medicine and Pain Management, Turku University Hospital and University of Turku, Turku, Finland

^132^ Department of Neurosurgery, Kaunas University of Health Sciences, Kaunas, Lithuania

^133^ Intensive Care and Department of Pediatric Surgery, Erasmus Medical Center, Sophia Children’s Hospital, Rotterdam, The Netherlands

^134^ Department of Neurosurgery, Kings college London, London, UK

^135^ Neurologie, Neurochirurgie und Psychiatrie, Charité – Universitätsmedizin Berlin, Berlin, Germany

^136^ Department of Intensive Care Adults, Erasmus MC– University Medical Center Rotterdam, Rotterdam, the Netherlands

^137^ icoMetrix NV, Leuven, Belgium

^138^ Movement Science Group, Faculty of Health and Life Sciences, Oxford Brookes University, Oxford, UK

^139^ Psychology Department, Antwerp University Hospital, Edegem, Belgium

^140^ Director of Neurocritical Care, University of California, Los Angeles, USA

^141^ Department of Neurosurgery, St.Olavs Hospital, Trondheim University Hospital, Trondheim, Norway

^142^ Department of Emergency Medicine, University of Florida, Gainesville, Florida, USA

^143^ Department of Neurosurgery, Charité – Universitätsmedizin Berlin, corporate member of Freie Universität Berlin, Humboldt-Universität zu Berlin, and Berlin Institute of Health, Berlin, Germany

^144^ VTT Technical Research Centre, Tampere, Finland

^145^ Section of Neurosurgery, Department of Surgery, Rady Faculty of Health Sciences, University of Manitoba, Winnipeg, MB, Canada
