# Supplementary material for: The burden of traumatic brain injury from low-energy falls among patients from 18 countries in the CENTER-TBI Registry: A comparative cohort study
Source: PLoS Med. 2021 Sep 14;18(9):e1003761. doi: 10.1371/journal.pmed.1003761 (PMC8509890; doi:10.1371/journal.pmed.1003761)
Supplement: Attachment — Submitted filename: CENTER TBI REGISTRY PAPER RESPONSE TO REVIEWERS.docx [file pmed.1003761.s018.docx]

-----------------------------------------------------------
Requests from the editors:

1. Competing Interests: Please add this statement to the manuscript's Competing Interests: "DM is an Academic Editor on PLOS Medicine's editorial board."

*The above statement has been added to competing interests*

2. Data availability statement: Please revise the statement. a) If the data are freely or publicly available, note this and state the location of the data: within the paper, in Supporting Information files, or in a public repository (include the DOI or accession number).

b) If the data are owned by a third party but freely available upon request, please note this and state the owner of the data set and contact information for data requests (web or email address). Note that a study author cannot be the contact person for the data.

c) If the data are not freely available, please describe briefly the ethical, legal, or contractual restriction that prevents you from sharing it. Please also include an appropriate contact (web or email address) for inquiries (again, this cannot be a study author).

We suggest revising the note regarding data access as: “Data cannot be shared due to XXX. Proposals to access the study data, data dictionary, analytic code, and analysis scripts may be submitted online at <https://www.center-tbi.eu/data>. Proposals are subject to review by the management committee. A Data Access Agreement is required, and all access must comply with regulatory restrictions imposed on the original study.” or similar.
*Data cannot be shared as the Consortium Agreement established between the CENTER TBI beneficiaries specifies the need for data access agreements with third parties^[1]^.*

*Thank you for the suggested modification, the data availability statement has been amended accordingly.*

3. Abstract: Please structure your abstract using the PLOS Medicine headings (Background, Methods and Findings, Conclusions).

*The abstract has been restructured with PLOS Medicine headings*

4. Abstract: Please quantify the main results (with 95% CIs and p values). In the last sentence of the Abstract Methods and Findings section, please describe the main limitation(s) of the study's methodology.

*We have reported all 95% CIs and p values in Findings.*

*We have added “The purposive sampling strategy and censorship of patient outcomes beyond hospital discharge are the main study limitations.” in “methods”.*

5. Abstract: Conclusions: Please address the study implications without overreaching what can be concluded from the data; beginning the section with the phrase "In this study, we observed ..." may be useful.
 *Thank you we have amended the abstract conclusion*

*“We observed that patients sustaining TBI from low energy falls are an important distinct demographic cohort; further that energy transfer level does not predict intracranial injury or acute care mortality in patients presenting to hospital. This suggests that energy transfer level should no longer inform prehospital and emergency department TBI triage in older people.”*

6. Author Summary: Thank you for including the summary box. Please reformat this into a short, bulleted, non-technical Author Summary of your research to make findings accessible to a wide audience that includes both scientists and non-scientists. The Author Summary should immediately follow the Abstract in your revised manuscript. This text is subject to editorial change and should be distinct from the scientific abstract. Please see our author guidelines for more information on Author Summary format: <https://journals.plos.org/plosmedicine/s/revising-your-manuscript#loc-author-summary>

*Thank you for the very helpful example we have revised the author summary accordingly*

7. References: Please use square brackets for in-text citations, like this [1]. Please do not include spaces within brackets when listing multiple references [1-4].

*The text referencing citations have been amended accordingly*

8. Line numbers: Please include line numbers throughout text when re-submitting the document.

*The resubmitted manuscript contains line numbers throughout the text*

9. Methods: Page 7: Please include in the text, the 17 countries included. If possible, please include the information/distributions for the 56 participating centres as supporting information.

*We have listed the 56 participating centres as supporting information (table viii), study approvals do not permit identification of patient recruitment by named centre. The revised supplementary material document contains graphical plot rates of low energy TBI by centre (supplemental/ figure i) . Please also see our response to point 11 re clustering.*

10. Methods: Page 8: It would be helpful to have more detail on the nature of the data collected: “We collected variables describing demographics (age & gender), pre-existing health status (<https://www.asahq.org/standards-and-guidelines/asa-physical-status-classification-system>), mechanism of injury, injury severity descriptors (Glasgow Coma Scale (GCS), Abbreviated Injury Scale (AIS)), presenting physiological vital signs, CT Brain findings, processes of care, and immediate outcome of care in terms of hospital mortality and destination on discharge (Tables 1-4). These variables were derived from the Utstein trauma template used for standard trauma registry collection across Europe, North America and Australasia.”

*We have expanded on these in the methods text listing in full the variables collected concerning pre injury health status (medication), presenting physiological vital signs, CT brain findings, processes and outcomes of care.*

11. Methods: Page 9: Please note how you accounted for clustering at the country level (and center within country).

*The original submission contained figure 2 showing rates of low energy TBI by country. The revised supplementary material document contains a graphical plot of prevalence of low energy transfer within the TBI cohort by centre (supplemental figure i). We have added the following text within the “strengths and limitations” subsection of “discussion”.*

*Figure 2 illustrates a considerable low energy TBI disease burden across countries - the median (IQR) prevalence of low energy transfer by centre was 36 (24-50)% (supplemental figure i) suggesting our findings do not result from clustering of low energy patients in a few large centres or in specific countries. Indeed, as one might expect the highest recruiting centres had lower rates of patients injured by low energy transfer – as study centres with larger catchment populations receive a greater proportion of their patients with TBI through EMS prehospital triage which prioritises high energy transfer [7,9].*

12. Methods: Page 9: Please explicitly indicate which analyses were done using chi-square, and which were done using Mann-Whitney tests, at least in the Table legends where the results are presented. And please clarify if all the continuous variables were non-parametric.

*This has been clarified in the legends of tables 1-4 in the resubmitted manuscript. The distributions of age, time intervals from arrival to imaging and interventions, Glasgow Coma Scale(GCS), Abbreviated Injury and Injury Severity scores (AIS, ISS) were non parametric – the latter 3 are ordinal scales.*

13. Methods: Did your study have a prospective protocol or analysis plan? Please state this (either way) early in the Methods section.

a) If a prospective analysis plan (from your funding proposal, IRB or other ethics committee submission, study protocol, or other planning document written before analyzing the data) was used in designing the study, please include the relevant prospectively written document with your revised manuscript as a Supporting Information file to be published alongside your study, and cite it in the Methods section. A legend for this file should be included at the end of your manuscript.

b) If no such document exists, please make sure that the Methods section transparently describes when analyses were planned, and when/why any data-driven changes to analyses took place.

c) In either case, changes in the analysis-- including those made in response to peer review comments-- should be identified as such in the Methods section of the paper, with rationale.

*Thank you we have inserted the paragraph below into methods*

*“An increased understanding of the traumatic brain injury disease burden through improved classification and identification of effective care are core objectives in the 2015 CENTER TBI published protocol of the core and registry studies. The protocol also specified the patient characteristic, care pathway and outcome variables for collection within the registry that could best support these objectives .^23^ The study objectives and analysis plan for determining the low energy TBI disease burden were stimulated by publications[3,6,7,18] highlighting falls as an increasingly common mechanism causing TBI - during and shortly after completion of CENTER TBI registry patient enrolment. The analysis plan was agreed by authors at a study meeting - University of Antwerp January 2019. We pre specified the energy transfer patient classification - informed by the literature ( figure 1); variables and statistical methods for comparison by energy transfer level (drafts of tables 1-4). The study analysis plan has not been modified since.”*

*This is confirmed in the transparency statement which is retained from the original manuscript (line 574).*

14. Methods: Page 10: Please revise the STROBE statement to: "This study is reported as per the Strengthening the Reporting of Observational Studies in Epidemiology (STROBE) guideline (S1 Checklist)."

*The statement has been revised.*

15. Results: Page 11: Please define the abbreviations “PM” and “TARN” at first use.

*We have inserted the definitions (TARN is also defined in the statistical analysis section (“methods”)*

16. Results: Page 11: Please clarify if the “Overall Cohort” results described and Table 1 pertain to the overall registry, or specifically those included in the study.

*In the revision we clarify that the overall cohort referred to in the text and in tables 1 and 2 combines the patients injured by both high and low energy transfer mechanisms.*

17. Results: Please provide 95% CIs and p values for all analyses described in the text.
*These are provided in the revised submission.*

18. Results: Page 18: The multivariable logistic regression here did not seem to be described in the Methods section. For the following result, please indicate the variables used in the adjustment:

“The 50% reduction in likelihood of critical care provision for the low energy cohort persisted after demographic, injury and comorbid considerations were accounted for in multivariable logistic regression - Adjusted Odds Ratio (AOR) 0·46 (95% Confidence Interval (CI) (0·43-0·50), a reduction in the likelihood of hospital admission was also observed for patients injured by low energy transfer albeit less so in older people. In patients with TBI admitted to the ward and intensive care the characteristics of patients injured by low energy transfer ( older age, pre-injury comorbidity and anticoagulation, non-evacuated mass lesion – Marshall VI- in ward admissions) were strong independent predictors of hospital mortality; after adjustment for these low energy transfer did not
independently predict mortality (suppl tables iv-vii); non-evacuated intracranial mass lesions were present in 3·1% (n=118) and 1·2% (n=53) of ward admission patients injured by low and high energy transfer.”

*The multivariable regression analyses were stimulated by the main study findings so had not been prespecified in the analysis plan. We precis this in the text prior to describing the results of the modelling in the final results paragraph.*

*“The observed differences in rates of critical care admission and the four fold greater “in hospital” mortality rate in patients with low energy TBI admitted to the ward were unexpected and prompted exploratory assessment of the contribution of energy transfer level to likelihood of critical care/ hospital admission and mortality - in risk adjusted analyses that were not pre-specified”*

*In the revision final results paragraph we have as requested detailed the variables used in the AOR from the multivariable model predicting intensive care admission (suppl table v).*

19. References: Please use the "Vancouver" style for reference formatting, and see our website for other reference guidelines <https://journals.plos.org/plosmedicine/s/submission-guidelines#loc-references>
*Thank you for the guidance we have modified the referencing accordingly*

20. Table 1: Please define all abbreviations in the legend (TBI, IQR).

*This has been done*

21. Supporting information files: Please include both a descriptive title and legend for each figure/table of the supporting information. In particular, please provide a description for Supporting Information Figure S2.

*Apologies if this was not clear in the original submission. Each figure and table has an adjacent descriptive title. The revised supplementary information now lists each in a legends section at the end.*

22. Supporting information Tables S4, S5, S6, S7: In the legend please provide the variables used in the adjustment. Please also present the unadjusted results.

*The revised legends lists the variables used in the adjustment.*

23. STROBE Checklist: Please reformat the checklist, using section and paragraph numbers to refer to locations within the text, rather than page numbers.

*This has been amended*

Comments from the reviewers:

Reviewer #1: The authors presented a well-constructed study addressing TBI Disease Burden focusing in low energy falls as mechanism of injury using data from the CENTER TBI registry. Whilst the data are interesting, they are very similar to those shown by a huge amount of studies evaluating geriatric TBI (actually, those patients who undergone low energy falls).

*Thank you for the supportive comment we agree with the review that there is some overlap between “geriatricTBI” and low energy TBI given the vulnerabilities associated with age. We focussed upon the energy transfer distinction within this study because of the lack of priority given to TBI from low level falls within EMS triage and injury prevention practice and the knowledge gap concerning disease burden.*

Since the study underscores that patients with low energy falls receive less invasive monitorization/treatments, it would be interesting to know how many of these patients had any limitation of life sustaining therapies. This would help to better characterize the population.

*We agree that this is an important consideration for further studies. The CENTER TBI Registry CRF did not record ceiling of care decisions. We acknowledge this at the end of the fourth paragraph of discussion*

*“The registry variables did not include “ceiling of care”, hence it is uncertain whether the apparent non-intervention strategy for the low energy cohort reflects a nihilistic approach.”*

*And in the conclusion:*

*“Further studies should test the justification for providing lower rates of critical care and emergency intervention for those injured by low energy mechanisms.”*

Minor comments:
Some references must be updated.
References are presented in different formats
 *The older references underpin currently used injury scoring systems [10,12,13,14] the reference formats have been amended for consistency*

Reviewer #2: "Traumatic Brain Injury Disease Burden from low energy falls: Findings of the CENTER TBI Registry" describes a comparative cohort study on the CENTER-TBI registry, involving over 21,000 patients presenting with traumatic brain injury (TBI) and indications for CT brain scan enrolled from 18 countries, from 2014 to 2018. The effect of various patient characteristics (both unadjusted and adjusted) is examined, for three clinical care pathways - emergency department (ED), admission to hospital but not ICU (ADM), and admission to the ICU (ICU). The main conclusion was that patients sustaining TBI comprise an important distinct demographic cohort, and that the level of energy transfer should not inform triage since it does not predict intracranial injury severity or threat to life.

While the scale of the registry and study is impressive, there are a number of issues that might be addressed:

1. Given the main recommendation of the study ("level of energy transfer should not inform triage"), it might be clarified as to the degree (if any) that level of energy transfer currently informs triage in the participating countries/hospital, cited guidelines ([8],[9]) notwithstanding.

*All hospital emergency departments in Europe use triage on patient arrival to determine priority for clinician review/imaging [8,9] – informed by injury energy transfer mechanism and patient vital signs. EMS clinicians similarly triage injured patients at scene in a similar way as indicated by our provider profiling of EMS in CENTER TBI [35]. EMS pre-alert hospitals of the imminent arrival of “triage positive” injured patients so that clinicians (trauma team) are available to provide resuscitation and prompt imaging.*

*35 M.C. Cnossen,1,ⱡ R. van der Brande,2, ⱡ H.F. Lingsma,1 S. Polinder,1 F. Lecky,3 A.I.R. Maas,2 and the CENTER TBI investigators and* $\text{participants. Prehospital}$*participants. Prehospital trauma care among 68 European neurotrauma centers: Results of the CENTER-TBI Provider Profiling Questionnaires. J Neurotrauma. 2018 Jul 24.  doi: 10.1089/neu.2018.5712. Online ahead of print.*

*We have added this a reference in “discussion”.*

- In particular, while Table 1 affirms that level of energy transfer does not strongly predict intracranial injury severity (at least as measured by GCS), it can be observed that the care pathway distribution differs significantly between high and low energy TBI (24.4% ICU for high, versus 11.5% ICU for low). However, it is not certain whether this difference in clinical pathway is due to triaging on level of energy transfer in the first place, or due to other criteria. Indeed, it is mentioned that "most published TBI series do not differentiate high from low energy transfer falls" (Page 6). This might be clarified.

*Thank you the reviewer highlights two important issues. We first address the reviewer’s second point which arises from previous studies utilising ICD mechanism of injury coding of hospital patients on discharge in administrative datasets rather than the information on mechanism that is more granular in the clinical setting and informs EMS/ED patient triage.[20] . The latter consideration has informed the CENTER TBI Registry CRF which records fall height - enabling the low energy TBI disease burden to be characterised in a pan European cohort.*

*The second point concerns the low rate of critical care for the low energy cohort which was unexpected given the similar rates of intracranial injury and overall mortality. We raise the concern of this possibly not being appropriate – but as the reviewer points out there may be explanatory factors other than triage, hence we conclude this is an area of concern for further study.*

2. Additionally, one might suspect that injury events that are classified as high energy transfer (e.g. traffic collisions, falling from a height, etc) may be more likely to be triaged as ICU, at least partly due to accompanying critical injury to other organs/limbs (e.g. internal injury to core organs, broken arms/legs). It is unclear whether these possible non-brain/head injuries were record/accounted for in adjustments, from the patient characteristics presented in Table 1, despite possibly being a major factor in triage outcomes in practice. This might be clarified.

*The reviewer makes a good point; Table 2 suggests that the severity of extracranial injuries is greater in the high energy cohort but still usually mild (median IQR extracranial ISS 5(1-13) versus ISS 2(0-8) P<0.001). We explored this and other potential confounders influencing critical care admission through modelling that was not prespecified in our analysis plan - described in the last paragraph of “results” ( supplemental table v). We found that after adjustment for the greater extracranial injury severity and other patient factors there was still a significantly lower rate of critical care provided to patients with TBI in the low energy cohort.*

3. On Page 7, the Inclusion/Exclusion criteria might be made more consistent with Figure 1. In particular, it is stated that "...the Registry included patients with pre-existing cognitive impairment etc... these three groups were exclused from recruitment to the CENTER TBI core study", while Figure 1 appears to describe the bulk of the exclusions (1101 patients) as "missing and unknown mechanisms of injury". Terminology might be standardized between the main text and the flowchart figure, and it might be made more explicit in the figure as to what the registry includes (the initial 22,849 patients?) and where the recruitment to the core study is complete (the 21,681 patients?)

*In the revision we clarify in methods (end of inclusion/exclusion paragraph) that the CENTER TBI core study is a separate smaller cohort (n=4509)of different patients enrolled from the same study centres. This is described in our published protocol of the core and registry studies [23]. The core cohort has greater representation of TBI patients admitted to critical care, more granular data and has been separately described[24].*

4. More generally, the existing triage criteria for assigning a patient to one of the three clinical pathways (ED, ADM, ICU; Page 8) might be explained in greater detail (partly alluded to in Point 1). If clinicians considered level of energy transfer as a criteria for triage, was it a major or minor criteria?

*As we indicate in the introduction triage results in allocating a higher level of care to patients who are “triage positive” using discriminators – such as energy transfer level - thought likely to indicate the presence of life changing injuries [3,8,9]. Triage occurs at the scene of injury and on arrival at hospital before the precise injuries are known (prior to CT scan) and those with the highest priority are more likely to have emergency departments pre-alerted to their arrival and/ or be given a higher priority on arrival and hence receive earlier/more senior medical review and CT scan (table 3). In some systems triage also influences selection of hospital that the patient is conveyed to from the injury scene.*

*Decisions on the care pathway for patients after the Emergency Department CT scan (decisions on discharge from hospital, or level of care for admitted patients) depend on imaging findings as well as the initial triage priority as the reviewer has identified on point 2.*

*Please also see our response to the reviewer’s first point.*

5. The purposive sampling procedure (Page 7) might be described in greater detail. How was it designed to produce a representative sample?

*In the revised methods section we state
“Sampling was purposive; training was provided so that study teams should include a representative sample of patients with TBI presenting within specific 24 hour periods ( with the selected 24 hour periods covering equally the different weekdays and seasons of the year).”*

6. On Page 8, the ED clinical pathway is described as "either discharge or direct to the mortuary from the ED"; it might be clarified as to whether "discharge from the ED" in this case, is a full discharge from the hospital, or possibly to general admissions/the ICU.

*The revised manuscript now states (lines 177-179)*

*“Patients were described in terms of one of three clinical care pathways after presentation which was either discharge* ***home*** *or direct to the mortuary from the Emergency department (ED),…”*

7. More details on the multivariate analyses (as presented in Supplementary Tables V to VII) might be provided. In particular, how were the variables involved for each of these analyses selected, given that some available variables from Tables 1 & 2 appear to be omitted? p-values might also be included.
emphasize selection

*The revised results section clarifies the selection of these variables*

*“The 50% reduction in likelihood of critical care provision for the low energy cohort persisted after variables usually influencing critical care admission decisions were adjusted for: demographic (age, gender), injury (ED arrival GCS /intubation status, ED pupillary responses, Marshall classification of CT findings, AIS grading of extracranial injury severity >2) and comorbid (pre-existing health and anticoagulation status”*

- *And*

*“In patients with TBI admitted to the ward and intensive care the characteristics of patients injured by low energy transfer (older age, pre-injury comorbidity and anticoagulation, non-evacuated mass lesion – Marshall VI- in ward admissions) were strong independent predictors of hospital mortality; after adjustment for these (and injury severity variables predicting hospital mortality after TBI [18])….”*

8. For EDGCS in Supplementary Tables V to VII, it appears presented as a binary category despite involving multiple groups/classes (as described in Table 1; contrast the Marshall Classification groups, which are analyzed individually). As such, the delineation of these groups/classes into a binary categorization might be described and justified.

*In supplemental table iv we have utilised a binary classification of GCS as < 15 versus 15 as in practice only patients who are back to baseline (GCS 15) are usually discharged home from hospital. For the other models concerning admission to critical care and prediction of mortality we utilise more granular information from GCS as per hospital mortality prognostic models in the TBI literature [18]. GCS is an ordinal scale reflecting severity of injury (scores from 3= unresponsive to 15=fully alert and orientated with normal responses).*

9. Still on Supplementary Tables V to VII, an AUC value (e.g. AUC = 0.90) is mentioned for each table in its caption. If these AUCs pertain to each assumed logistic regression model in predicting ICU/mortality, it might be considered to also present the corresponding ROC curves.

*ROC curves are now provided in supplemental information figures with the AUC values iii-vi*

10. A general reservation is that while the manuscript suggests that TBI triage on energy transfer does not inform risks of intracranial injury/hospital mortality and thus should be discarded, it does not appear to propose and justify a concrete alternative. In particular, the presented multivariate analyses would seem to have the potential to quantitatively suggest an alternative triage model (or risk score, since it is admitted in the limitations section that the analyses are post-hoc; therefore, the impact of improved triage is probably difficult to estimate). This might be considered by the authors.

triage on vital signs more obvious threat to life, abandon energy transfer for older people

*The major impact of de-emphasising energy transfer as a TBI triage discriminator would be that patients with low energy transfer would be given equivalent priority to those with high energy transfer if their vital signs (the other important component of triage tools [8,9] were similar).*

11. There are a number of phrasing issues, e.g. the sentences "Injury classification by energy transfer mechanisms..."; "Patients injured by high energy mechanisms being conveyed..." (Page 5), "Forty percent (8622) of patients..." (Page 15), "significant longer term rates of disability..." (Page 22), etc.

*Thank you we have amended and hopefully improved the phrasing highlighted by the reviewer*

Reviewer #3: Markus Skrifvars, University of Helsinki
Regarding the paper "Traumatic Brain Injury Disease Burden from low energy falls: Findings of the CENTER TBI Registry" submitted to PLOS Medicine. This a sub-study of the large TBI registry CENTER-TBI. This paper contains data from a large database/study with potential interest to the general medical audience. I have the following comments:

1. The major aim of this study is to compare low and high energy trauma. Therefore it is paramount to include more precise information on how this classification was done. Perhaps include a Table with the different types of in the two groups? Was this classification done on site? Was there any data validation between centers in order to make sure that different centers scored this the same way? Of course one may debate whether a low velocity car accident or bicycle accident (with a helmet) is different from a fall from standing were the energy on the head may be substantial.

*Our real world study utilised registry methodology extracting variables as described in the patient’s clinical record i.e. the energy transfer mechanism recorded by clinical staff that informed triage and subsequent care. In “limitations” we state:*

*“The reality of injury incidents being unwitnessed - particularly in people falling whilst alone at home - means prehospital staff estimate fall height resulting in possible misclassification; there is also some variation in classification of the height above ground that is considered as low energy ^3,8,9^”*

2.      The manuscript mentions that patients with a suspected or confirmed TBI who had a CT brain done were included. Did there need to be a clear presented history of a trauma? I refer to the elderly patient who is found at home without any clear evidence of what has happened. Continuing on from this do you have information on how many of the abnormal CT brain findings were unexpected? Again many of these patients will have an unclear event history with many factors making the diagnostic work up more difficult (intoxiocations, seazures, cardiac arrhythmias etc.).

*The reviewer highlights some of the important challenges in the diagnosis of TBI. We have separately studied imaging practice in study centres which is liberal in this context [1] allowing for unexpected findings.*

3.    One would intuitively think that the use of "not for ICU", DNR and other treatment limitations would impact the admission of the elderly patients with multiple comorbidities to ICU. Is this information included in the registry?

*We agree with the reviewer that this is a salient consideration for further studies. The CENTER TBI Registry CRF did not record ceiling of care decisions. We acknowledge this at the end of the fourth paragraph of discussion*

*“The registry variables did not include “ceiling of care”, hence it is uncertain whether the apparent non-intervention strategy for the low energy cohort reflects a nihilistic approach.”*

*And in the conclusion:*

*“Further studies should test the justification for providing lower rates of critical care and emergency intervention for those injured by low energy mechanisms.”*

- 4.      I would personally like to see the results of a multivariable model predicting outcome including high/low energy. This could even be done for only the patients with an abnormal CT scan. The key question is, if you look at two similar abnormal CT scans will the outcome be different if they are the result of a high or low energy? When you have the abnormal CT in front of you what are the factors that drive admission to ICU? I suspect it is age.

*We have addressed this with the post hoc analyses described in the final paragraph of “results” in the manuscript. Likelihood of ICU admission (supplemental table v) is adjusted for the factors suggested by the reviewer. At the end of results we report* 

*“The 50% reduction in likelihood of critical care provision for the low energy cohort persisted after variables usually influencing critical care admission decisions were adjusted for: demographic (age, gender), injury (ED arrival GCS /intubation status, ED pupillary responses, Marshall classification of CT findings, AIS grading of extracranial injury severity >2) and comorbid (pre-existing health and anticoagulation status”*

- *And*

*“In patients with TBI admitted to the ward and intensive care the characteristics of patients injured by low energy transfer (older age, pre-injury comorbidity and anticoagulation, non-evacuated mass lesion – Marshall VI- in ward admissions) were strong independent predictors of hospital mortality;*

5.  Continuing on from the previous point. I would be interested to know if one ends up in the ICU does it then make any difference for outcome (survival, neurological outcome) if the energy was high or low?

*As per the reviewer’s previous point we address this in supplemental table vii. Essentially the factors associated with low energy TBI – particularly, pre-existing health, anticoagulation status and age, are strong predictors of hospital mortality and explain the higher low energy crude mortality rates alongside the more traditionally accepted TBI severity predictors (GCS, pupillary responses, Marshall CT grading).*

6.  Do you have information about frailty and functional capacity? Again I suspect that this piece of information will drive the decision on further care.

*We have recorded the American Society of Anaesthesiology pre injury health status, anticoagulation medication and age (Table 1). These are adjusted for in the aforementioned model of critical care admission and mortality*

7.    In the first sentence of the discussion there is a statement that 40% of patients with TBI have a low energy injury pattern. Does this comparison also include those with a negative CT brain? Perhaps state more specifically about numbers in those with a CT brain confirmed TBI?

*We found that the rate of positive CT brain scans to be similar in high (32%) and low energy (29%) cohorts (table 2) hence the low energy proportion of abnormal CT brain scans (2520 /6746 = 37%) is similar to the low energy proportion of the overall cohort (40%).*

8.  You mention in the discussion that the low-energy patients may be receive less intense care. This may be the case but is this the results of the injury being of low energy or the fact that these patients are not perceived to benefit from these interventions? Unless you present data that many of these "low energy" patients experience late deterioration with emergency surgery and ICU admission this may not be completely supported by your data.

*We describe in the results text and in table 3 that times to critical care admission were similar in the high and low energy cohorts (153 and 150 minutes after study hospital arrival. Also in the results text (line 341) we record that*

*“the craniotomy rate was greater in the low energy cohort (4·2% (95% CI 2.2-6.3%) versus 3·1% (95% CI 1.4-4.8%), P-value <0·001). (Table 2). In the low energy cohort there was a greater time delay between arrival at the study hospital and the provision of emergency interventions such as craniotomy (Table 3).”*

*We stand by our findings that the low rate of critical care provision for the low energy cohort is a concern requiring further study - given the equivalent rates of intracranial injury and hospital mortality in the energy transfer cohorts. Please also see our response to the reviewer’s third point*

9.    The European map with differences in incidence of low energy TBI is intriguing. However, this is not discussed in the manuscript. Were the centers included in the CENTER-TBI study of comparable enabling the drawing of such a Figure? Indeed one would like to see the equivalent of a 95% confidence interval in the colors. This is of course not possible but somehow certainly of these findings must be presented.

*In the original submission we commented in results*

*“the prevalence of low energy transfer varied from 30% to 50% in most participating countries (figure 2)”*

*The revised supplementary material document contains a graphical plot of prevalence of low energy transfer within the TBI cohort by centre (supplemental figure i) which we now also refer to in the results text. We have added the following text within the “strengths and limitations” subsection of “discussion”.*

*“Figure 2 illustrates a considerable low energy TBI disease burden across countries - the median (IQR) prevalence of low energy transfer by centre was 36 (24-50)% (supplemental figure i) suggesting our findings do not result from clustering of low energy patients in a few large centres or in specific countries. Indeed, as one might expect the highest recruiting centres had lower rates of patients injured by low energy transfer – as study centres with larger catchment populations receive a greater proportion of their patients with TBI through EMS prehospital triage which prioritises high energy transfer [7,9].”*

10.  Do you have information on how many patients were intoxicated? Again data suggests the association between alcohol/drugs and TBI from low-energy trauma.

*Although the median age of the low energy cohort was 74 years with most injuries occurring at home (table 1) we agree with the reviewer that intoxication may have contributed to low energy falls and also to high energy transfer TBI ( assaults and road traffic incidents). We have added the following sentence to the discussion of study strengths and limitations*

*“The registry did not record acute alcohol/drug ingestion which may contribute to both energy transfer cohorts (low energy falls, assaults and road traffic collisions) “.*

11. In the discussion the authors state that anti-platelet medication effects cannot be reversed. How the administration of platelets?

*We refer to the recently published PATCH trial that suggested platelet transfusion does not benefit patients taking anti platelet medication with intracranial bleeding. We have added this as a reference. [33]*

12. The conclusion includes some ideas about the need for future studies especially regarding the need for more studies on the intensity of TBI care in low-energy cohort. Do we really think that it is the injury energy in itself that is driving care decisions and not the patient? I suspect that most clinicians make their decision on a combination of things with patient age, comorbidities and functional status being in focus. So unless the authors have compelling data that really challenges this, perhaps the conclusion should be softened.

*Our non prespecified analysis (supplemental table v) suggests that the lower intensity of care for the low energy cohort is not entirely explained by age, comorbidity and injury severity however we regard this analysis as exploratory and have tempered our conclusion accordingly*

*“Further studies should test the justification for providing lower rates of critical care and emergency intervention for those injured by low energy mechanisms.”*
