# Supplementary material for: The burden of traumatic brain injury from low-energy falls among patients from 18 countries in the CENTER-TBI Registry: A comparative cohort study
Source: PLoS Med. 2021 Sep 14;18(9):e1003761. doi: 10.1371/journal.pmed.1003761 (PMC8509890; doi:10.1371/journal.pmed.1003761)
Supplement: Attachment — Submitted filename: PLOS MED Responses to Editor and Reviewers.docx [file pmed.1003761.s019.docx]

Reviewer 2

Reviewer #2: We thank the authors for addressing the previously raised points, and concerns over potential confounding on severity of extracranial injuries with reference to Supplemental Table V, which appears also updated with univariate odds from the previous revision (as is Supplemental Table IV).

*We are glad the previous revision and response addressed the reviewer’s initial requests. We have addressed their comments on the revision below.*

1. It might however be explained as to why some values in the (updated) Supplemental Table V, appear different from the previous revision. For example, adjusted odds ratio for Significant Extracranial Injury is now 1.60 (1.40-1.82), while previously it was 1.59 (1.40-1.81). It might also be considered to explicitly state the baseline reference and condition being varied, for these tables (e.g. baseline being low energy, versus high energy)

*In this second revision we now explicitly state the reference categories for binary categorical variables in suppl tables iv-vii (sex, energy transfer, intubation status on ED arrival, significant extracranial injury). For significant extracranial injury AIS3+ the reference standard is “No significant extracranial injury AIS <3”.*

*The reviewer correctly identifies small changes between our original and revised submissions in AORs. These represent correction of minor transcription errors from the R studio model outputs that were in the original submission - for which we apologise. The corrections have not changed reporting or interpretation of these exploratory findings in “Results”.*

2 The revised Results section states that "The 50% reduction in likelihood of critical care provision for the low energy cohort persisted after variables influencing critical care admission decisions were adjusted... Adjusted Odds Ratio (AOR) 0·46 (95% CI 0·43-0·50) - AOR 0.77 (95% CI 0.53-1.12) after accounting for the interaction between age and energy transfer mechanism (supplemental table v)". However, it is not clear where the initial 0.46 OR arises from, in the actual Supplemental Table V. The relevant univariate OR appears to be given as 0.36. This might be clarified.

*The Adjusted Odds Ratio (AOR) 0·46 (95% CI 0·43-0·50) arises from multivariable model predicting ICU admission the suppl table v When the model omits the interaction of age/ energy transfer as a predictor the estimated AOR is 0.46 and this is now stated within the revised suppl table v legend.*

3. The additional ROC curves provided as requested are appreciated. However, Supplemental Figure IV shows an AUROC = 0.9012, while its corresponding Supplemental Table V gives AUC = 0.91, which does not appear to correspond after rounding, unlike the other three figure/table pairs. This might be clarified.

*Thank you for identifying this typographical error. In this revision we have corrected the suppl table v legend accordingly (AUC 0.91).*

4. It has been partly clarified that "Decisions on the care pathway for patients after the Emergency Department CT scan (decisions on discharge from hospital, or level of care for admitted patients) depend on imaging findings as well as the initial triage priority". Further specific details on the care pathways might be described, if possible.

*In “Introduction” we describe the role of energy transfer mechanism informing triage at the injury scene and on Emergency Department arrival;*

*“Patients injured by high energy transfer mechanisms being conveyed to higher levels of care within specialist Trauma centres.”*

*In this revision we have added further detail to the pathway description at the end of “Methods” in “Case Report Form (CRF) Variables”*

*“Patients were described in terms of one of three clinical care pathways after presentation, triage and CT brain scan; discharge home or direct to the mortuary from the Emergency department (ED), admission to the hospital but not to the ICU (ADM), or admission to the Intensive Care Unit (ICU).”*

5. The explanation for Point 8 on EDGCS as a binary category, might also be included in the manuscript or supplementary material in some capacity.

*We have added this to the “suppl table iv” legend.*

Reviewer #3: Markus Skrifvars University of Helsinki
Regarding the revised version of the paper "Traumatic Brain Injury Disease Burden from low 2 energy falls: Findings of the CENTER TBI Registry" submitted to Plos Medicine. The authors have revised their manuscript. The paper contains important information about the epidemiology if TBI care. However some of the conclusions made and recommendations are not completely supported by the data and further softening is in my mind required. From a societal/research perspective the key issue is the need to include these patients in TBI trials. My personal clinical take from this teh very risky patient group of; the elderly patient using anticoagulants/antiplatelet agents with low energy fall.

*We appreciate the reviewer’s comments concerning the importance of our findings and address their further observations below*

1. -       In some parts of the manuscript I feel that there still is a suggestion that "low energy" in itself is the main reason why the patient does not receive ICU care For example on line 408-409 the authors state "However, the low energy cohort was 50% less likely to receive critical care (12% versus 24%) or emergency intervention (7·5% versus 13%)". I would suggest that one could also use the word "need" instead of "receive". I do not think that a study such as this can provide solid evidence that it is the injury mechanism in itself that prompted the neurosurgeon/neurointensivist NOT to admit the patient.

*We appreciate the reviewer’s interest concerning the observed lower provision of critical care to the low energy cohort – despite the equivalence of some parameters that correlate with clinical need (rates of hospital admission, mortality, and abnormal CT scan). However, in “Discussion” we indicate that further research is indicated concerning need for critical care;*

*“The registry variables did not include “ceiling of care”, hence it is uncertain whether the apparent non-intervention strategy for the low energy cohort reflects a nihilistic approach.”*

*AND*

*“Further studies should test the justification for providing lower rates of critical care and emergency intervention for those injured by low energy mechanisms.”*

2. -       Continuing from the previous comment, what do the authors suggest we should do in case of a low energy TBI patient with a GCS 14-15 patient and with a non-operatively managed injury on the CT scan? What is the ICU intervention that will make a difference?

*Thank you we agree this is not a traditional ICU population in many centres although 11.5% of our low energy cohort did receive critical care. This TBI population has a high incidence of one or more pre-injury health issues (table 1). . Decompensation of pre-existing conditions may cause low falls. Therefore, patients in this TBI population may require complex management of both co-morbidities and their traumatic injury including close monitoring, stabilisation and consideration of organ support. Further, some therapies for pre-existing conditions - such as anticoagulation therapy which is 3 times more prevalent in the low energy cohort – require reversal or modification in the context of TBI (table 1, figure 3).*

*These inferences are supported by the exploratory supplementary modelling where comorbidity has a stronger influence on hospital mortality in the ward admission compared to the ICU cohort (suppl tables vi and vii). In “Discussion” we comment;*

*“These findings point to the need for a holistic personalised medicine approach to TBI patients requiring; multidisciplinary acute care. Such an approach addresses each patient’s pre-existing health issues, the specific brain injury sustained and their interaction.”*

3.  As the authors are comparing high and low energy falls it is quite clear that the reason the high energy group may have needed ICU care not due to their TBI but to other injuries. For example, how many were sedated/mechanically ventilated in the ED? It is quite clear that such a patient will go to the ICU no matter what?

*In “Results” we note the greater requirement for intubation in the high energy cohort due to the higher rates of moderate and severe TBI on presentation to hospital and intubation prior to hospital arrival (tables 1 and 2). We also note the higher requirement for extracranial injury intervention (table 2). We do not suggest a reduction in critical care provision for a multiply injured patient with TBI. Our findings suggest there is potential to expand the lower level of critical care /organ support provided for patients injured by low energy transfer subject to further research (please also see our response to the reviewer’s first two points).*

4. The statement in conclusion "This suggests energy transfer level should no longer inform prehospital and emergency department TBI triage in older people". This is a bit problematic I think. I think one can say based on these findings that a low energy level does not rule out significant TBI with high morbidity and mortality. But on the other hand, I do think that a significant injury mechanism must still be seen as a major contributor to morbidity and mortality and should treated as such. There is also the risk of multi-trauma. I would maintain that from the pre-hospital standpoint an elderly patient with a low energy fall is more likely to have a mild TBI/other pathology than one injured for example in a high speed traffic collision.   

*We have amended this statement in view of the reviewer’s concerns:*

*“This suggests energy transfer level may not be relevant to prehospital and emergency department TBI triage in older people.”*

*Injury triage is based on presenting physiology (GCS and vital signs including active bleeding from extracranial wounds)) and energy transfer considerations. Our study suggests – contrary to current guidance (9) - that energy transfer may not be relevant to TBI triage in the majority of patients who present with TBI who have GCS 13-15 and stable vital signs (tables 1 and 2) particularly older people. Patients with TBI who are GCS <13 and/ or have abnormal vital signs – are currently given a high triage priority in the context of both high and low energy transfer (9).*

5.  It would be very interesting to the see the IMPACT risk in the high and low energy cohorts. In a simple model with the IMPACT risk included is the trauma energy a significant predictor of outcome? Is this the same in different age categories and based on use of antiplatelet agents.

*We again thank the reviewer for their interest in our findings. We have not used the IMPACT model (22) in our exploratory analyses (suppl tables iv-vii) as it was not developed for prediction of hospital mortality and is not applicable to patients with mild TBI. IMPACT;*

*-       is recommended for use in patients with GCS < 13 81.6% of our study cohort have GCS 13-15 (Table 1)*

*-       was derived and validated on studies that often excluded patients with TBI over 65 years.*

*-       Mainly studied prognosis with regard to 6 month functional outcomes or mortality at 6 months*

*Our exploratory analyses utilised variables shown to predict hospital mortality in patients with TBI (all severities and ages) in more recent studies (Helmy et al ref 18).*

6.The authors mentions alcohol and drug intoxication as a challenge for pre-hospital TBI care. Can the authors perhaps provide a reference for this and slightly expand what they mean? Are intoxicated patients thought to have a more severe TBI or vice versa

*The challenge for prehospital triage is the attribution of GCS<13 in patients who are intoxicated prior to sustaining head injury - potential causes being the intoxicant and/ or TBI. Current guidance advises against attribution of low GCS (and a lower triage priority with delayed CT brain scans) solely to alcohol or drugs based on underpinning research (8,9).*

Editor comments:

1. Please completely address the points of the reviewers.

*Thank you for the opportunity to address these further points in the above responses and those made by the editors below.*

2. Data availability statement: Please remove the initial “Data sharing statement:” from this section.

*This is now removed*

3. Title: Please revise your title according to PLOS Medicine's style. Your title must be nondeclarative and not a question. It should begin with main concept if possible. "Effect of" should be used only if causality can be inferred, i.e., for an RCT. Please place the study design ("A randomized controlled trial," "A retrospective study," "A modelling study," etc.) in the subtitle (ie, after a colon).
We suggest: “Traumatic brain injury disease burden from low energy falls among patients from 17 countries from the CENTER-TBI Registry: A comparative cohort study” or similar, mentioning the design and setting.

*The title is now amended to be consistent with PLoS Medicine style.*

*“Traumatic Brain Injury Disease Burden from low energy falls: A comparative cohort study of patients from 18 countries - the CENTER TBI Registry”*

*(c.f. “Methods” The CENTER TBI Registry included Israel and 17 European Countries)*

4. Abstract: Please combine the Methods and Findings into a single section, with the main limitations as the final sentence of this section.

*Methods and Findings have been combined as advised above.*

5. Abstract: Line 61-62: Please reword this sentence if possible, to clarify.

*This sentence has been reworded.*

6. Abstract: Lines 65-76: Where the findings are reported, it is not completely clear that the first group is the low energy and the second is the high energy cohort. Please clarify this if possible.

*The revised abstract contains acronyms for low and high energy (LE and HE) to clarify the reported figures in parenthesis.*

7. Abstract: Line 71-72: Please clarify whether the finding of CT abnormalities was or was not significantly different between groups (p<0.001 is reported, where the text reports the groups were similar).

*The reporting of this finding has been clarified as below.*

*“The CT brain scan traumatic abnormality rate was 3% lower in the low energy cohort (LE= 29% (95% CI 27-31%) versus HE = 32% (95% CI 31-34%) p<0.001)”*

*The difference is statistically but not clinically significant. This is expanded upon in “Discussion – Strengths and Limitations”*

8. Abstract: Line 81-82: We suggest tempering this conclusion, given some of the limitations pointed out by the reviewer. “This suggests energy transfer level should no longer inform prehospital and emergency department TBI triage in older people.”

*Thank you for the suggestion. In line with our response to reviewer 3 (point 4) we have tempered this*

*“This suggests energy transfer level may not be relevant to prehospital and emergency department TBI triage in older people.”*

9. Author Summary: Please remove the “summary box” and please format using bullet points (we suggest 3-4 for each of the three sections). The third section should be titled “What do these findings mean?”

*The box has been removed and the author summary formatted as suggested*.

10. Author Summary: Please temper the second and third points of the “What do these findings mean” section, please interpret the study based on the results presented in the abstract, without overstating your conclusions.

*These have been amended accordingly;*

*“-In older people the assumption that energy transfer predicts brain injury severity and threat to life appears to lack validity.*

*-Our findings suggest that energy transfer considerations may not be relevant to Emergency Medical Services triage of older patients with TBI.”*

11. Methods: Analysis Plan: Thank you for describing the analysis plan for your study. If feasible, please include a document version of the analysis plan as a supporting information file.

*CENTER TBI Registry data were available immediately after the study meeting agreeing the analysis in January 2019. Hence the analysis plan document from March 2019 (uploaded supplemental file) includes early drafts of tables and figures*.

12. Results: For the “Comparisons by Energy Transfer mechanism” and “Injury characteristics” sections, please make it more clear in the text where possible, the nature of the two groups being compared (for example, where two sets of values refer to the low and high energy transfer groups, respectively).

*The revised sections contain acronyms for low and high energy (LE and HE) to clarify the reported figures in parenthesis.*

13. Results: Line 314-317: Please clarify if these differences reached statistical significance (Table 2 seems to suggest they may). “In patients injured by low energy falls the proportion with abnormalities detected on CT scan (including skull fracture) and proportion of abnormal scans showing intracranial injury (29% (95% CI 27-31%) and 70% (95% CI 69-73%) was similar to that of patients injured by high energy mechanisms (32% (95% CI 31-34%) and 75% (95% CI 74-77%)).

*The significant p value has now been inserted and the text amended*

*“was lower than that of patients injured by high energy mechanisms”*

14. Results: Line 342-344: Please clarify that for this finding, this was significant only for specific intervention (craniotomy) as shown in Table 3. “In the low energy cohort there was a greater time delay between arrival at the study hospital and the provision of emergency interventions such as craniotomy (Table 3).”

*This has now been clarified. The greater delay in the provision of emergency interventions to the low energy cohort was clinically significant for all 4 emergency intervention groupings in table 3. However, the low numbers requiring interventions other than craniotomy meant reduced power to demonstrate statistically significant time differences (type 2 error).*

15. Results: Line 371-378: Please revise this sentence to clarify, or split into multiple sentences.

*This has now been split into multiple sentences.*

16. Discussion: Line 406: Please clarify this sentence to reflect the study findings. In your analysis, there seemed to be a significant differences in CT abnormality between the two groups, for example. You note later in the limitations section that these may not be clinically meaningful, but perhaps that could be expanded on/clarified here.

*We have revised to state the rates of CT abnormality are “clinically similar” which is consistent with a statistically significant difference.*

17. Conclusions: Line 511: Please modify this sentence with “...to our knowledge, the largest…” or similar wording.

*This has been modified to include “to our knowledge”*

18. Conclusions: Line 515: Please revise this sentence to temper the recommendations somewhat: Our findings suggest that within the older cohort, TBI triage based on energy transfer may not inform risk of intracranial injury and hospital mortality.”

*The sentence has been tempered as suggested.*

19. Acknowledgements: Please remove the funding information from this section, and ensure all information is accurately entered into the Financial Disclosure and Competing Interests section of the manuscript submission form.

*The funding information has been removed from the manuscript document and is retained as advised within the manuscript submission form.*

20. Page 28-29: Please remove the “Financial Disclosure” and “Competing Interests” sections from the main text of the manuscript- this information should be accurately entered into the manuscript submission form.

*This has been done.*

21. Line 583: List of The CENTER-TBI participants and investigators. Please include those who contributed to the work but do not meet our authorship criteria should be listed in the Acknowledgements with a description of the contribution. Authors are responsible for ensuring that anyone named in the Acknowledgements agrees to be named.

*This is a listing of Group Contributors which this revision now places within the Acknowledgements section. All those listed have agreed to the listing which is inherent to all CENTER TBI publications. The Group contributors include local Investigators and CENTER TBI grant beneficiaries (23).*

22. Figure 1: Please include a descriptive legend, including a definition of RTC.

*Figure legends including acronym definitions are uploaded as a supplemental file.*

23. Figure 2: Please confirm that the appropriate usage rights apply to the use of this map. Please see our guidelines for map images: <https://journals.plos.org/plosmedicine/s/figures#loc-maps>

*The map used was from*

[*https://en.wikipedia.org/wiki/File:Europe_blank_map.png*](https://en.wikipedia.org/wiki/File:Europe_blank_map.png)

*It is a free to use map which was edited using Photoshop for different colours and gradients.*

[*https://en.wikipedia.org/wiki/File:Europe_blank_map.png#file*](https://en.wikipedia.org/wiki/File:Europe_blank_map.png#file)

24. Figure 3: Please include a legend, including definitions for all abbreviations used.

*Figure legends including acronym definitions are uploaded as a supplemental file,*

25. Table 2: Please clarify ASH vs ASDH in the table/legend.

*This has been done.*

26. Checklist: Please remove all references to page numbers.

*These have been removed.*
