# Supplementary material for: The burden of traumatic brain injury from low-energy falls among patients from 18 countries in the CENTER-TBI Registry: A comparative cohort study
Source: PLoS Med. 2021 Sep 14;18(9):e1003761. doi: 10.1371/journal.pmed.1003761 (PMC8509890; doi:10.1371/journal.pmed.1003761)
Supplement: Attachment — Submitted filename: Responses to Requests from Editors.docx [file pmed.1003761.s020.docx]

1. Title: We suggest revising the title to: The burden of traumatic brain injury from low energy falls among patients from 18 countries in the CENTER TBI registry: A comparative cohort study”

***Thank you this has been revised accordingly***

2. Data availability statement: Please remove the opening set of quotation marks (“Data cannot be shared…).

***These have been removed***

3. Abstract: Lines 59-60: We suggest reformatting this sentence to clarify, and incorporating it into the following paragraph: “22782 eligible patients were enrolled, and study outcomes were analysed on 21681 TBI patients with known injury mechanism.”

***The sentence has been reformatted accordingly in the revision.***

4. Abstract: Line 72-73: Please clarify this sentence to make it clear it is the low energy group who were more likely to not receive critical care and emergency intervention.

***This has now been amended in the revision***

***“…; the low energy cohort were 50% less likely to receive critical care (LE =12% (95% CI 9.5-13%) versus HE=24% (95% CI 23-26%) p<0.001) or emergency interventions (LE=7·5% (95%CI 5.4-9.5%) versus HE=13% (95%CI 12-15%) p<0.001) than patients injured by high energy transfer.”***

5. Abstract: Line 79-81: We suggest revising to “...and further, our findings suggest that energy transfer level may not predict intracranial injury or acute care mortality in patients with TBI presenting to hospital. This suggests factors beyond energy transfer level may be more relevant to prehospital and emergency department TBI triage in older people.” or similar, depending on your meaning.

***Thank you this section of the abstract has been amended as suggested above.***

6. Author summary: Line 91: We suggest revising “are given lower priority” to “can be given lower priority” as it seems like this wouldn’t universally be the case depending on the circumstances.

***This has been amended as suggested above.***

7. Author summary: Line 101: We suggest revising to: “We found that the 40% of patients with TBI who were injured through low energy falls were significantly older…”

***This has been amended as suggested.***

8. Author summary: Line 109-110: We suggest revising to “Low energy falls contribute to a significant portion of the TBI disease burden, which will increase…”

***This has been amended as suggested.***

9. Author summary: Line 113-114: We suggest clarifying the wording here that the findings don’t necessarily indicate that fall energy level is not relevant to the triage process.

***Thank you. We have amended this to be consistent with the amendment in to the abstract conclusion (point 5 above);***

***“Factors beyond energy transfer level may be more relevant to prehospital and emergency department TBI triage in older people”***

10. Introduction: Line 124-126: Please clarify to indicate that this is not an all-inclusive list of the sources of high and low energy transfer mechanisms “(High energy transfer resulting from road traffic collisions, falling from a height, blunt assault or contact sports while low energy transfer results from low level falls or those from a standing height [6,7,8])”

***Thank you – to this effect we have inserted “most commonly” before “resulting” in the revision.***

11. Introduction: Line 127-128: We suggest revising to: “informing Emergency Medical Service (EMS) on scene trauma triage [3,8,9], with patients injured by high energy transfer…”

***This has been amended accordingly.***

12. Methods: Line 208: Please clarify if the energy mechanism classification list here is inclusive of all types of injuries presented.

***The revision clarifies that patients injured by “other” less common high energy transfer incidents were included. Lines 212-213***

***“…., sports and other high energy transfer incidents were classified as high energy TBI….[6-8]”***

13. Results: Line 334: Please fix the “s” typo in this sentence: “...was s lower than that of patients”

***Apologies for the typo which has been corrected.***

14. Results: Line 352: Please check if an end parenthesis is missing from: “(median (IQR) extracranial ISS; LE = 2 (0-8) versus HE = 5 (1-13) p<0.001.”

***The missing end parenthesis has been inserted.***

15. Results: Line 381-384: Please clarify the numbers associated with the high energy group in this sentence: “...6% higher in ICU patients (22% (95% CI 17-28%) versus 16 % (95% CI 13-20%)) 383 and four times greater in admitted patients (4.2% (95% CI 1.1–7.3%) versus 0·9% (95% CI 0.0 -3.8%)...”

***This has been clarified.***

16. Discussion: Line 407-408 and at Line 425: We suggest making the distinction between European hospitals and hospitals located in Israel. Please similarly adjust the language at line 410-411- “the first pan European study” as it is not clear if Israel is being considered as a European country.

***The language has been adjusted to make the distinction (Line 412 and Line 415).***

17. Discussion: Line 411-413: Please revise this sentence to clarify if you mean that there are a greater proportion of older adults in the low energy fall cohort or among all of those presenting with TBI: “Compared to the broader past literature we observed a greater proportion of older adults (>65 years old)-almost 39% as opposed to 10-17% previously reported elsewhere [22,27].”

***This has been clarified and now reads***

***“Compared to the broader past literature we observed a greater proportion of older adults (>65 years old)- almost 39% of all patients presenting with TBI – as opposed to 10-17% previously reported elsewhere [22,27].”***

18. Discussion: Line 457: Please remove the superscript formatting applied to the word “and” in the sentence.

***The formatting has been removed.***

19. Discussion: Line 476: Please clarify, here and throughout, if there are 18 or 17 countries represented in the study.

***There are 18 in total – 17 in Europe plus Israel – this has been clarified throughout.***

20. Conclusions: Line 515-517 Please also change to “across Europe and Israel” or similar.

***This has been changed.***

21. Page 33: Please remove the “Role of the funding source” section from the main manuscript, and ensure the information is accurately entered into the Financial Disclosures section of the manuscript submission system.

***This has been removed***

22. Page 33: Please remove the “Data sharing” section from the main manuscript, and ensure the information is accurately entered into the Data Availability section of the manuscript submission system.

***This has been removed***

23. Acknowledgements: We suggest listing all contributing members of the group, with affiliations, in a supporting information file. Or, including the names in the acknowledgements, but moving the affiliations to a supporting information file.

***We have moved the affiliations to a supporting information file S17 Appendix***

24. Figure 1: Please remove the extra paragraph symbols from the figure. If possible, please increase the font size, as it is difficult to read. Please include a brief descriptive legend. Please indicate the original sample of 22849 as those enrolled in the registry, and please clarify (here and or the first sentence of the results) why the number is different from the 22782 mentioned in the Results as enrolled in the Registry.

***The extra paragraph symbols have been removed***

***The font size has been increased***

***The figure contains a descriptive legend and a list of the main figure legends has also been separately uploaded file S18.Appendix.***

***Figure 1: Identification of patients injured by high and low energy transfer. (RTC=Road Traffic Collision)***

***We have amended the first sentence of results to include the figure 22849:***

***“Fifty-six study centres from 18 countries (17 European Countries and Israel) enrolled 22849 patients to the CENTER TBI Registry; (for cumulative recruitment over the study see figure in S1_fig). A total of 21681 TBI patients - with known clinical care pathway and injury mechanism - were included in the overall cohort for analysis (figure 1);***

25. Figure 2: We suggest increasing the font size, if possible. Please also include a legend explaining the color map as it relates to low energy injury prevalence. Please note in the legend:

***The font size has been increased. The figure contains a key which explains the colour shading and a legend which reads***

***Figure 2: Prevalence of low energy as TBI causal mechanism in countries participating in the Center TBI Registry – (colour shading as per key. Map from*** [***https://en.wikipedia.org/wiki/File:Europe_blank_map.png***](https://en.wikipedia.org/wiki/File:Europe_blank_map.png)***)***

***A list of the main figure legends has also been separately uploaded file S18 Appendix..***

26. Figure 3: Please include a descriptive legend that describes what is illustrated in the figure.

***The full descriptive legend is now contained within the figure***

27. Analysis Plan: We suggest renaming this file “S1 Analysis Plan” or similar. If possible, please note in the document that this describes a prospectively developed analysis plan for the project (as written it seems as if it is a preliminary report on the findings).

***The document Analysis plan records analysis planned at the study meeting and preliminary findings as data were available. It is not possible to amend the analysis plan without changing the document from March 2019. However in Methods (Lines 224-226) we explain***

***“The analysis plan was agreed by authors at a study meeting - University of Antwerp January 2019. We pre specified the energy transfer patient classification - informed by the literature (figure 1); variables and statistical methods for comparison by energy transfer level (drafts of tables 1-4) (S2 Analysis plan).”***

28. Supporting information files: Supplementary tables: Please provide a “clean” version of the document without markup. It was not clear which table went with which title/legend from tables 4-8. Please include the title and legend with each table individually.

***We have created separate clean supplemental files for each table each containing the table legend***

29. Table 1: We suggest “no pre-existing medical conditions” or similar instead of “normal healthy patient” in the left column under Pre-Injury Health Status.

***The classifications arise from the American Society of Anaesthesiology Classification which were used in the Registy crf, this uses the classification “normal healthy patient”. Hence we feel this should be retained in Table 1.***

***https://www.asahq.org/standards-and-guidelines/asa-physical-status-classification-system***

30. Supporting information files naming: You may use almost any description as the item name of your supporting information as long as it contains an "S" and number. For example, “S1 Appendix” and “S2 Appendix,” “S1 Table” and “S2 Table,” and so forth.

Please use whole numbers when naming your supporting information files. Combine separate parts (e.g., S1A and S1B Table) into one file (e.g. S1 Table) or rename with whole numbers (e.g., S1 and S2 Table).

Please match the names of your supporting information files with the supporting information captions within your manuscript. For example, a PDF file for “S2 Fig.” must be named “S2_fig.pdf”.

If you wish to refer to an element within a supporting information file, such as a table within a supporting text file, cite it in one of the following ways: “Table A in S1 Text,” “Table in S1 Table,” or “data in S1 Text.” Please do NOT cite it as “S1 Table in S1 Text.” This may lead to hyperlinking errors.

***We have renamed all supplemental files as separate tables figures and text - in the order that they are cited in the manuscript and amended their citation as advised.***
